# Phylogeny-aware comparative genomics of *Vibrio vulnificus* links genetic traits to pathogenicity

**DOI:** 10.1128/mbio.00205-26

**Published:** 2026-06-17

**Authors:** Luis F. Delgado, David J. Riedinger, Víctor Fernández-Juárez, Daniel P. R. Herlemann, Christian Pansch, Marija Kataržytė, Greta Gyraitė, Jens Hulterström, Thorsten B. H. Reusch, Marcin Rakowski, Kasia Piwosz, Adam Woźniczka, Heike Benterbusch-Brockmöller, Theodor Sperlea, Susann Dupke, Holger C. Scholz, Sandra Kube, Lasse Riemann, Matthias Labrenz, Anders F. Andersson

**Affiliations:** 1 KTH Royal Institute of Technology, SciLifeLab, Stockholm, Sweden; 2 Leibniz Institute for Baltic Sea Research Warnemünde (IOW) 28389, Rostock, Germany; 3 University of Copenhagen 4321, Helsingør, Denmark; 4 Estonian University of Life Sciences 85334, Tartu, Estonia; 5 Åbo Akademi University 1040, Turku, Finland; 6 Klaipėda University, Klaipėda, Lithuania; 7 Vilnius University 54694, Vilnius, Lithuania; 8 GEOMAR Helmholtz-Zentrum für Ozeanforschung Kiel, Kiel, Germany; 9 National Marine Fisheries Research Institute 123876, Gdynia, Poland; 10 Robert Koch Institute 9222, Berlin, Germany; National Institutes of Health, Bethesda, Maryland, USA

**Keywords:** *Vibrio vulnificus*, comparative genomics, phylogenomics, virulence, pathogenicity

## Abstract

*Vibrio vulnificus*, a coastal marine bacterium and opportunistic pathogen, poses increasing health risks due to rising sea water temperatures. While it harbors diverse virulence factors, its disease mechanisms remain poorly understood. To address this, we sequenced 82 environmental isolates from the Baltic Sea and combined them with 325 published genomes (clinical and environmental) for comparative analysis. Phylogenetic reconstruction revealed four major lineages, with Baltic strains restricted to L2 and L4. Clinical and environmental strains were found across all lineages, suggesting phylogeny reflects environmental adaptation more than pathogenicity. Using our newly developed PhyloBOTL pipeline, we identified 128 orthologs enriched in clinical isolates, grouped into 36 co-localization clusters. These include both known and novel virulence candidates, such as chaperone-usher pili, type VI secretion effectors, and spermidine synthases. Some clusters showed convergent loss across clades, implying niche-specific evolution. We also designed PCR primers targeting these genes to aid in the surveillance of pathogenic *V. vulnificus* strains.

**IMPORTANCE:**

*Vibrio vulnificus* is a naturally occurring marine bacterium that can cause life-threatening infections, with incidence increasing as coastal waters warm due to climate change. Determining which environmental strains pose the greatest risk remains a major challenge for public health surveillance. By analyzing hundreds of genomes from globally distributed strains, including extensive sampling from the rapidly warming Baltic Sea, this study shows that pathogenic potential is not restricted to specific evolutionary lineages but is instead associated with distinct sets of genes enriched in clinical strains. To support pathogen detection, we developed PCR primers targeting a subset of these clinically associated genes. Together, these findings provide new insight into the molecular basis of *V. vulnificus* infections and offer practical tools for improved detection, monitoring, and risk assessment of harmful strains in coastal waters and seafood under ongoing climate change.

## INTRODUCTION

*Vibrio vulnificus*, a gram-negative, rod-shaped bacterium within the *Vibrio* genus, naturally inhabits estuarine, coastal, and brackish waters with a near-global distribution (1, 2). This opportunistic pathogen, capable of causing severe and occasionally lethal infections, poses a significant threat to humans and is a prominent contributor to non-cholera *Vibrio*-related fatalities globally (3).

*V. vulnificus* can enter the human body through open wounds in the skin, with even minor wounds serving as potential points of entry (4). This can result in severe wound infections that may necessitate limb amputation and can progress to an overwhelming septic shock, frequently culminating in fatality due to multiple organ failure (5). Another transmission route is through the consumption of raw contaminated seafood, particularly oysters (6). The most susceptible populations for fulminant extraintestinal infections from either the oral or cutaneous route are individuals with compromised immune responses. This includes patients with thalassemia, diabetes, HIV, or liver diseases (cirrhosis or hepatitis) and those receiving immunosuppressant drugs for other underlying conditions (7, 8). A comprehensive review of 459 U.S. cases reported to the Food and Drug Administration (FDA) between 1992 and 2007 revealed a staggering 51.6% mortality rate among patients infected with *V. vulnificus* (9).

Infections associated with *Vibrio* spp. exhibit a correlation with elevated water temperatures surpassing approximately 15°C and a moderate salinity range of PSU 2–25 (10). Over the past decade, in tandem with the warming trends observed in the North and Baltic Seas, there has been a surge in reported cases of *Vibrio* infections in this region (11–13). Recent surveys of *V. vulnificus* in coastal zones of the Baltic Sea have confirmed the regulatory influence of temperature and salinity on the species, while also suggesting a potential role for eutrophication status (14, 15). Since the trend of increasing *Vibrio*-related infections and fatalities along the Baltic Sea coasts is expected to be worsened by climate change (16), thereby posing a significant threat to human health and the tourism industry, assays for fast and accurate detection of *V. vulnificus* in environmental as well as seafood samples are needed (17).

*V. vulnificus* harbors a wide array of virulence factors, including mechanisms for acid neutralization, capsular polysaccharide production, iron acquisition, cytotoxicity, motility, and adhesion (9). However, its precise disease mechanisms remain poorly defined. Mouse model studies have shown that not all *V. vulnificus* isolates have the same virulence potential (18, 19). However, unlike *Vibrio cholerae*, where all cholera-causing strains are affiliated with a single clade (20), genomic analyses of *V. vulnificus* have revealed that clinical strains exhibit extensive genomic diversity. Comparative genomics of clinical and environmental isolates makes it possible to identify key virulence factors and genomic signatures associated with pathogenicity. Since the first *V. vulnificus* genome (CMCP6) was sequenced in 2002 (21), more than 500 genomes have been made publicly available in GenBank, approximately 40% from clinical isolates and 60% from environmental sources. However, the majority of environmental isolates originate from animal reservoirs, primarily fish and shellfish. Only a limited number are derived directly from environmental matrices such as water and sediments, where selection pressure for maintaining virulence genes is likely lower than that in host-associated environments. While the growing availability of genome sequences has advanced our understanding of *V. vulnificus* population structure (22), phylogenetics (23, 24), and virulence (25–27), key aspects of its pathogenic mechanisms remain unresolved.

The limited representation of genomes from non-animal environmental sources remains a key gap in current resources, and the genomic diversity of *V. vulnificus* in the Baltic Sea region has been little explored. To address this, we sequenced and assembled genomes from 82 environmental isolates obtained from water, sediment, and seagrass along the Baltic Sea coast. We integrated these data with publicly available genomes from clinical and environmental sources worldwide and analyzed them using a newly developed comparative genomics pipeline, PhyloBOTL (Phylogeny-Based Ortholog–Trait Linkage). This framework combines ortholog identification, phylogenetic reconstruction, and phylogeny-aware gene–trait association analysis to identify genes whose gain or loss is associated with pathogenicity, while also grouping the associated genes into putative functional modules based on chromosomal proximity.

Using this approach, we identify candidate virulence-associated genes linked to clinical isolates. Finally, we leverage these findings to design PCR primers targeting clinically enriched genes, providing new tools for the detection and surveillance of potentially pathogenic *V. vulnificus* strains in environmental and seafood samples.

## RESULTS AND DISCUSSION

### Phylogenetic structure of *V. vulnificus*

**Fig 1:**
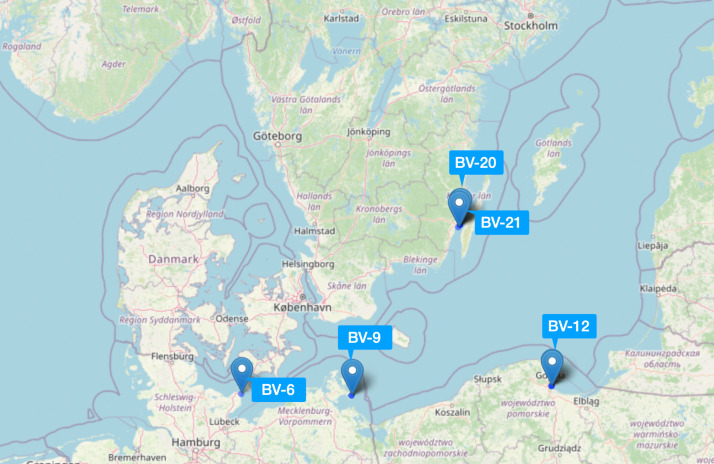
Sampling locations of the *V. vulnificus* isolates sequenced in this study. “BV” denotes BaltVib, the larger project this study was part of. The map was created with the leaflet package in R (28).

We sequenced the genomes of 82 *V. vulnificus* strains isolated from five different sites along the Baltic Sea coastline during the summer of 2021 (15). These sites encompassed two locations in Sweden, one in Poland, and two in Germany (Fig. 1; interactive map in File S1). The water temperature and salinity at the sites ranged from 16.5°C to 20.3°C and 6.5–10.4 PSU, respectively (Table 1). The strains were isolated from the water column, from the top sediment layer, and from eelgrass (*Zostera marina*) leaves, within or proximate to eelgrass meadows (Table S1 provides contextual data for the isolates). DNA sequencing and assembly yielded genomes ranging in size from 4,838,252 to 5,241,188 base pairs (bp) (Table S2). Seventeen of the strains harbored plasmids, ranging in size from 44,678 to 143,195 bp, and 53 were predicted to have prophage regions, ranging from 2101 to 120,375 bp. Several antibiotic resistance genes were detected in the 82 isolates. Among these, the genes *varG* and *tet(34)* are present in all, and *tet(35)* is only absent in six isolates. These genes confer resistance to various antibiotic classes, including carbapenem, tetracycline, and beta-lactam (Table S3).

**TABLE 1:** Sampling stations of the *V. vulnificus* isolates sequenced in this study^*a*^

Station	No. of isolates	Country	Avg water temp (°C)	Avg water salinity (PSU)
BV-21	1(w)	Sweden	16.5	6.7
BV-20	11(w); 9(s)	Sweden	16.8	6.5
BV-12	6(w); 47(s); 2(z)	Poland	20	7.0
BV-09	1(w); 2(s)	Germany	20.3	7.7
BV-06	3(s)	Germany	19.3	10.5

^*a*^ Isolation source: w, water; s, sediment; z, *Zostera marina* leaf. Additional contextual data are provided in Table S1.

**Fig 2:**
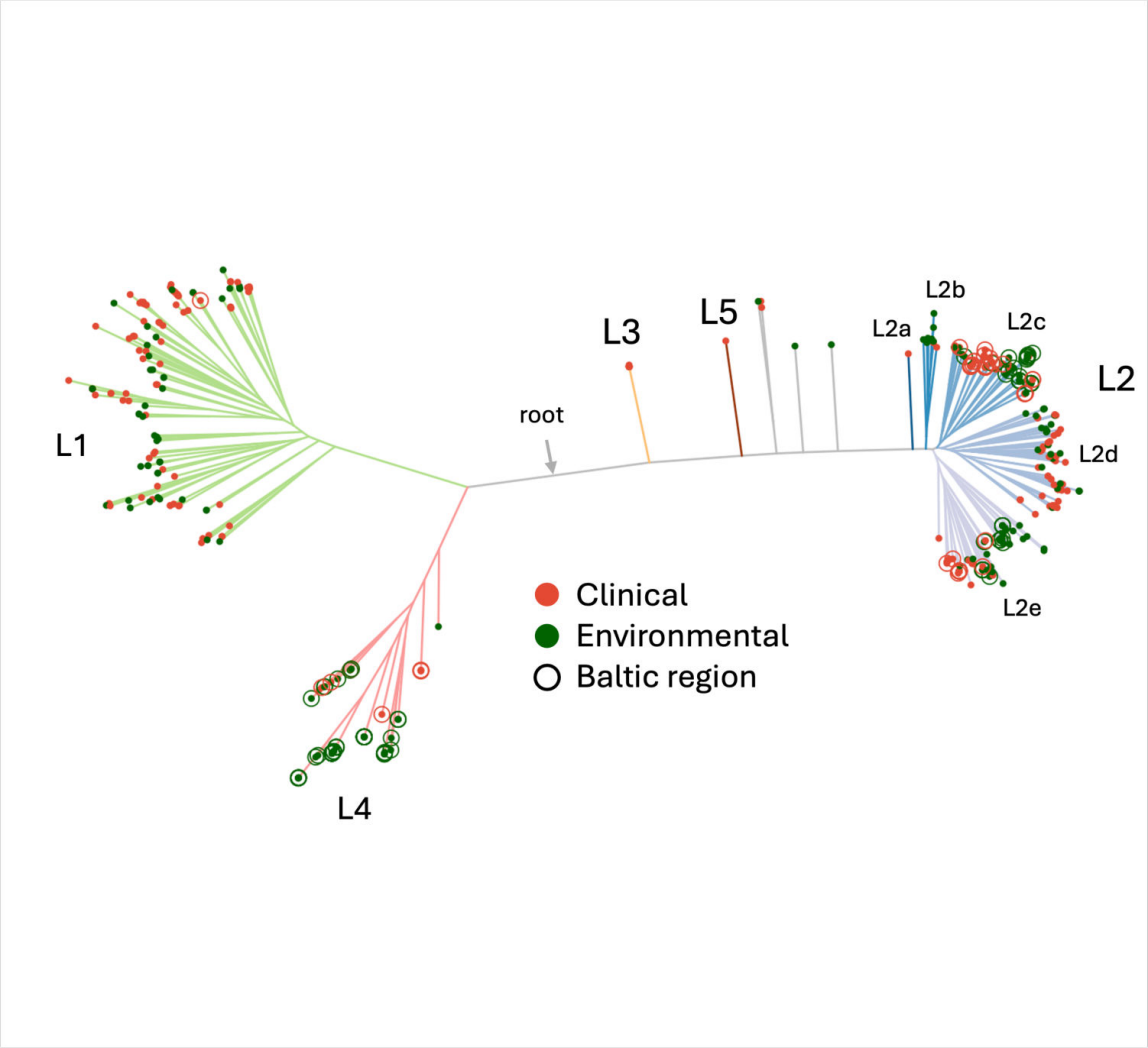
Maximum-likelihood phylogeny based on protein-coding regions of the core genome. Lineage numbers are according to Roig et al. (23), but the L2 sublineages (L2a–L2e) were introduced in this study. Branches are colored according to (sub)lineage. Leaf colors indicate clinical (*n* = 208) or environmental (*n* = 199) isolate source, and circles indicate Baltic Sea region isolates (*n* = 133, of which 82 were sequenced in this study). The approximate location of the root is indicated by the arrow and was determined in a separate phylogenetic reconstruction that also included other *Vibrio* species.

We complemented the 82 new genomes with 325 previously published environmental (*n* = 117) and clinical (*n* = 208) *V. vulnificus* genomes to facilitate a comprehensive comparative analysis (Table S2 provides metadata on the included genomes). Of the previously published genomes, 4 of the environmental and 47 of the clinical (mainly from the 2018 heatwave [11, 12]) stem from the Baltic Sea region (Germany, Denmark, Norway, Sweden, and Finland). The remaining genomes are primarily from the United States (*n* = 204), China (*n* = 26), Israel (*n* = 10), South Korea (*n* = 9), and Colombia (*n* = 9). We used maximum-likelihood to infer the phylogeny of the 407 strains, leveraging an alignment of 2,223,636 nucleotide positions from the core genome, with 361,414 positions exhibiting variation. Consistent with the findings of Roig et al. (23) and subsequently Lopez et al. (26), our study confirmed the formation of four major lineages (L1–L4) among the strains. Additionally, consistent with Roig et al., we identified a fifth lineage (L5) comprising a single genome. Furthermore, our expanded data set revealed three additional lineages, encompassing six genomes in total, positioned between L5 and L2 (Fig. 2).

Notably, all of the new 82 Baltic Sea genomes fall in the L2 and L4 lineages, which is also the case for the previously published 51 genomes from the Baltic Sea region (except for a single isolate; DSM 11507; for which the isolation source is unclear). Lineage L4 comprises a small number of previously published isolates from Germany and Sweden and two isolates from the Ebro Delta in Spain (23). The lineage is now greatly expanded, comprising 46 genomes from Sweden, Poland, and Germany. Lineage L2 is composed of five sublineages, which we denote L2a–L2e. Interestingly, the Baltic Sea region genomes are confined to sublineages L2c and L2e, collectively representing 61% of the genomes in these sublineages (Fig. 2).

The biased phylogenetic distribution of the Baltic Sea region genomes may suggest that the phylogenetic lineages of *V. vulnificus* constitute strains adapted to distinct environmental conditions. Temperature emerges as a plausible factor, since the cold winters in the Baltic Sea region may require specific adaptations not needed at lower latitudes with a warmer climate. However, the presence of multiple isolates from tropical regions in L2c and L2e contradicts the notion of these sublineages being exclusively cold-adapted. Salinity is another potential factor. In the central to southwestern Baltic Sea, salinity ranges from 6 to 14 PSU, while *V. vulnificus* can thrive in environments with considerably higher salinity. Due to the unavailability of data on salinity, temperature, and other physicochemical parameters for most published environmental genomes, a comprehensive phylogenomic assessment of these factors remains unfeasible at present. However, a recent study by Lopez et al. (26) of two sites in Florida indicates the importance of salinity. Specifically, six out of seven isolates from the site with higher salinity (29–32 PSU) clustered within lineage L1, whereas 11 out of 14 isolates from the site with lower salinity (6–19 PSU) clustered within L2. There was no significant temperature difference between the sites; hence, salinity appears more important in this case. However, the underlying drivers may be other environmental factors differing between the sites.

Since most human clinical isolates have earlier been found to cluster in lineage L1, this lineage has been proposed to carry an increased genetic potential for virulence (26). However, this view was recently challenged by a study sequencing a large number of clinical isolates from the US, where the isolates were found to be equally distributed between L1 and L2, although infections caused by L1 isolates demonstrated a borderline significant higher mortality (26). Our finding that both clinical and environmental isolates from the Baltic Sea region are confined to Lineage L2 and L4 reinforces that clinical strains are not restricted to L1 but instead suggests the phylogenetic structure of the species reflects environmental factors. Another potential explanation could be geographic barrier effects, i.e., that the sublineages are restricted to geographic regions due to dispersal limitation (22).

### Genes enriched in clinical isolates

The expanded number of genomes from environmental strains allows identifying genes associated with pathogenicity by comparing the gene content in the environmental and clinical isolates. Although many of the environmental isolates may well have pathogenic potential, we can assume that a fraction of them lack such capacity, and thus, genes that are significantly enriched in the clinical isolates are potentially of importance for the pathogenicity trait. We used a phylogeny-aware approach (29) that identifies genes (orthologs) whose presence co-evolves with a trait over the phylogenetic tree. Thus, orthologs that are gained (or lost) in conjunction with switching from the environmental to clinical “trait” more often than expected by chance over evolution will be identified. We identified 13,920 orthologous groups of genes (OGs, also sometimes referred to as “orthologs” in the following) among the 407 genomes. Among these, 128 were found to be enriched and 82 depleted in the clinical vs. environmental strains (false discovery rate-adjusted *P*-value < 0.05). The 128 clinically enriched orthologs had significantly more matches in the Virulence Factor Database (30) than orthologs found in at least 95% of the genomes (χ² test, *P*-value = 0.0035), supporting the strategy. In the following sections, we will focus on the orthologs enriched in the clinical strains.

**Fig 3:**
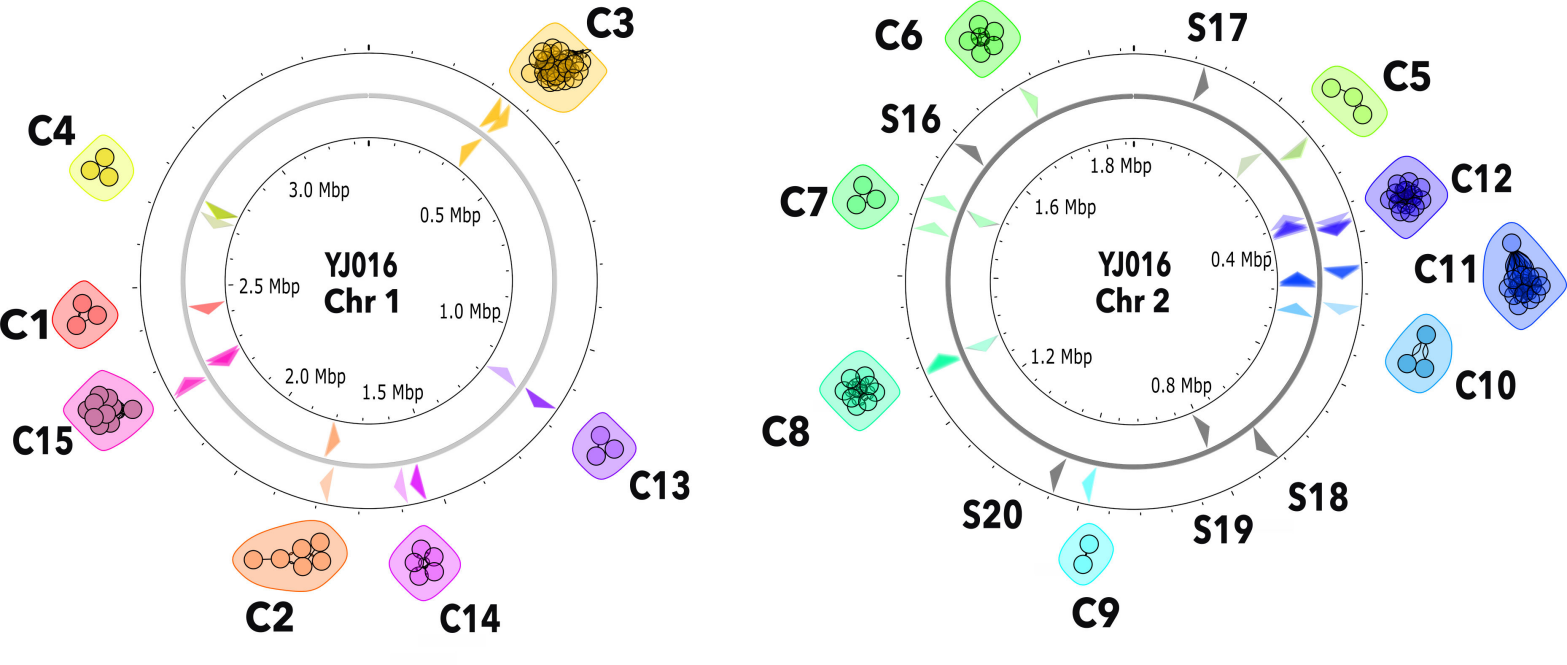
Co-localization clusters of clinically enriched orthologs. The orthologs’ positions and directionality in the two *V. vulnificus* YJ016 chromosomes are indicated with arrows, colored according to co-localization clusters. Gray arrows represent singleton clusters. Not all singleton clusters are present in strain YJ016.

**Fig 4:**
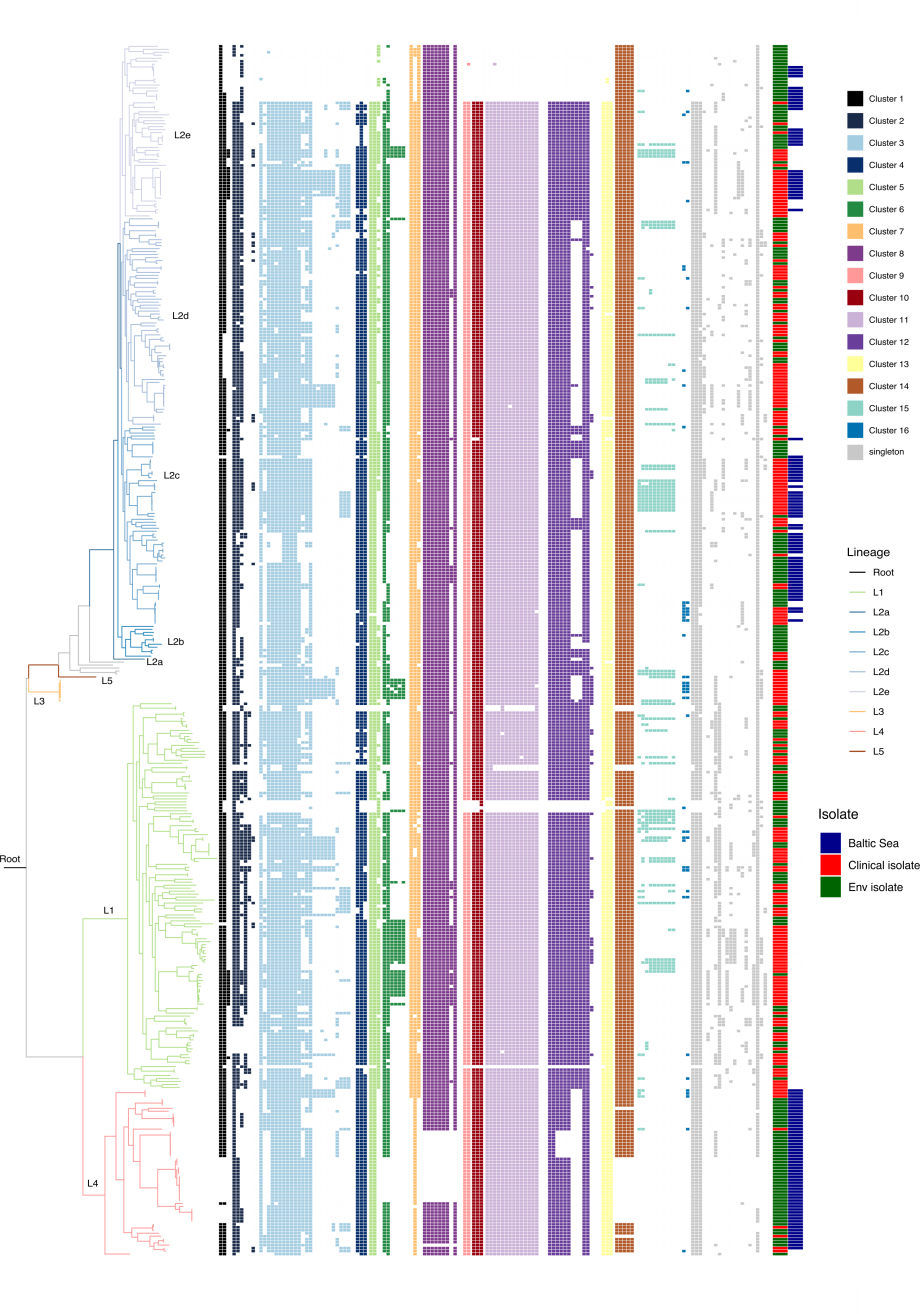
Presence of the 128 clinically enriched orthologs in the 407 *V. vulnificus* genomes. The orthologs (one per column) are ordered and colored according to co-localization cluster. The right-most columns indicate if the isolate is clinical or environmental and whether it is from the Baltic Sea region. The branches of the phylogenetic tree are colored according to (sub)lineage.

Since functionally related genes are often encoded in the same genomic region on bacterial chromosomes (31, 32), we used information on the orthologs’ pairwise proximity in the *V. vulnificus* genomes to cluster them (Materials and Methods). This resulted in 36 co-localization clusters, of which 20 were singletons and the remaining consisted of 2–24 OGs (Fig. 3 and 4; Table 2; detailed information in Table S4). Twenty-one of these were located on chromosome I, 14 on chromosome II, and one on a plasmid. The co-localization clusters entailed both those containing genes previously linked to pathogenicity in *V. vulnificus,* such as genes for biofilm formation and capsular polysaccharide synthesis, and clusters with genes that, to our knowledge, have not previously been associated with pathogenicity in the species. These include genes of the chaperone-usher pathway for pilus synthesis, genes for spermidine synthesis, genes encoding effector proteins of the type VI secretion system, and a gene for an RTX toxin-like protein. In the following sections, we provide a more detailed description of selected co-localization clusters, with the aim of encouraging follow-up studies to further investigate their potential roles in pathogenicity in *V. vulnificus*. In addition to our internal OG IDs, gene IDs for the corresponding genes in the CMCP6 strain are provided when the gene is present.

**TABLE 2:** Summary of co-localization clusters of clinically enriched orthologs^*a*^

Cluster	Predicted function	No. of OGs	Location
1	Chemotaxis	3	Chr. 1
2	Unknown	6	Chr. 1
3	Capsular polysaccharide (CPS) synthesis	24	Chr. 1
4	T6SS effector protein	3	Chr. 1
5	Adhesion (MSCRAMM family)	3	Chr. 2
6	Unknown	6	Chr. 2
7	Multidrug efflux pump	3	Chr. 2
8	ABC-type multidrug transport system	9	Chr. 2
9	Unknown	2	Chr. 2
10	Potential spermidine synthase	3	Chr. 2
11	Biofilm formation	14	Chr. 2
12	RTX toxin-like protein	12	Chr. 2
13	Chemotaxis	3	Chr. 1
14	Chaperone-usher pili biogenesis	5	Chr. 1
15	Tethrationate respiration	10	Chr. 1
16	Transposon	2	Plasmid

^*a*^ Predicted function refers to the function of the cluster as a whole or of an ortholog within the cluster. Only clusters with >1 ortholog are shown. See Table S4 for information on all clusters and individual orthologs.

Cluster 3 is the largest cluster, containing 24 enriched orthologs, located on chromosome I. This cluster includes several genes known to be involved in capsular polysaccharide (CPS) synthesis (33). CPS is considered the most important virulence factor of *V. vulnificus* (34). It mediates resistance to complement-mediated bacteriolysis and phagocytosis and is essential for the bacterium’s survival in human serum (35). Its expression has been demonstrated to undergo phase variation (36, 37), and acapsular strains have proven to be non-infectious (38). CPS is not required for biofilm formation in *V. vulnificus* (39), and its expression even inhibits biofilm formation (40). The CPS synthesis gene locus of *V. vulnificus* is similar to that of the group 1 or 4 capsules of *E. coli* (41). As shown in Fig. S1, the cluster is highly diverse, with the enriched orthologs found in 322 different gene order configurations, and never more than six isolates sharing the same configuration. Eleven of the orthologs are more prevalent than the other in the cluster, present in 92%–100% of the clinical and 70%–88% of the environmental strains. Among these are *wza-wzb-wzc*, responsible for polymerization control and translocation of CPS across the outer membrane (33), all of which are present in 352 out of 407 strains. Of the 55 strains where one or more is missing, only five are clinical, in line with CPS being a crucial virulence factor.

Cluster 11 comprises 14 orthologs situated in a genomic region on chromosome II involved in the formation of biofilm. The enriched orthologs include the genes of the *cabABC* operon, responsible for production and secretion of the calcium-binding extracellular matrix protein CabA, the *brpABCDFHIJK* locus, which is one of several loci responsible for assembly and export of exopolysaccharide (EPS) in *V. vulnificus*, and the regulatory genes *brpT* and *brpS* (42, 43) (Fig. S2). While EPS is the predominant component of the *V. vulnificus* biofilm matrix, CabA contributes to the structural integrity and robustness of the biofilms (44). The expression of *cabABC* and the *brp* locus undergoes regulation through a complex regulatory network involving BrpS, BrpT, and the master regulator BrpR, and is activated under conditions of elevated levels of the intracellular second messenger cyclic dimeric guanosine monophosphate (c-di-GMP) (39, 42, 45, 46). Although *V. vulnificus* encodes nearly 100 proteins predicted to synthesize, degrade, and bind c-di-GMP, relatively little is known about what environmental signals regulate c-di-GMP levels, and thereby biofilm formation (47). However, calcium was shown to be a primary environmental signal that specifically increases intracellular c-di-GMP concentrations, which in turn triggers biofilm formation (47). The calcium-induced biofilm formation is likely important for colonization and accumulation of *V. vulnificus* in oysters and other bivalves that have high concentrations of calcium on their surfaces (48). See further discussion on c-di-GMP in Appendix S1.

Cluster S16 includes a single ortholog (OG0003733; VV2_1694) that is functionally related to Cluster 11. The ortholog corresponds to *brpN,* an acyltransferase-3 domain-harboring protein recently found to be important for EPS production in *V. vulnificus* (49). Together with the above-described *cabABC* and *brpABCDFHIJK* loci, as well as a third locus for EPS synthesis—the *brpLG* locus (39, 42, 45, 46)*—brpN* is essential for the formation of robust biofilms and rugose colonies in *V. vulnificus*. Similar to the other loci, it is regulated by BrpT and ultimately by the master regulator BrpR (49).

Cluster 14 consists of five enriched orthologs, arranged as an operon on chromosome I. This operon includes a pilus assembly chaperone (OG0003726; VV1_2742), an outer membrane pilus assembly protein (usher) (OG0003709; VV1_2741), a fimbrial/pilus subunit protein (OG0003723; VV1_2740), a LuxR-family response regulator (OG0003721; VV1_2744), and a protein with an unknown function (OG0003722; VV1_2743) (Fig. S3). Alongside another operon in *V. vulnificus*, it has been categorized within the archaic σ-clade of the chaperone-usher (CU) pathway for pilus biogenesis (50). Although widely distributed among gram-negative bacteria, the structural makeup of these archaic pili has remained elusive until a recent study revealed their assembly into an ultrathin, superelastic spring structure (51). In *Acinetobacter baumannii* and *Pseudomonas aeruginosa*, σ-clade pili play a role in surface attachment and biofilm formation (52, 53). While the involvement of multiple type IV pilus systems in *V. vulnificus* is well-documented, contributing to biofilm formation, adherence to epithelial cells, and virulence (54, 55), the ecological significance and potential virulence of σ-clade pili, to our knowledge, remain unexplored in the species. However, in a gene expression study comparing normal and elevated c-di-GMP conditions, the fimbrial/pilus subunit gene (VV1_2744) was repressed in elevated c-di-GMP conditions (42), indicating that these pili are not involved in biofilm formation.

Cluster 4 includes three enriched orthologs on chromosome I: a diguanylate cyclase (OG0003715; VV1_1733), a PAAR repeat-containing protein (OG0003626; VV1_1702), and a protein annotated as permease in UniRef (OG0000002; VV1_1704). Diguanylate cyclase (DGC) synthesizes c-di-GMP. In most isolates, the DGC is encoded rather distantly from the other two orthologs (Fig. S4) and may not be directly functionally coupled to these. In contrast, the other two are mostly encoded adjacently, with OG0003626 upstream of OG0000002, sometimes with multiple copies of OG0000002 in series. OG0003626 encodes an N-terminal PAAR repeat followed by a MIX_IV signal. The MIX (Marker for type sIX) is commonly found in Type VI secretion system (T6SS) effector proteins that carry C-terminal toxins (56). T6SS are commonly found in Gram-negative bacterial genomes and are made up of 13 conserved proteins. T6SS has dual functions, secreting both anti-eukaryotic and anti-prokaryotic effectors. The PAAR repeat of OG0003626 provides binding to the VgrG protein that forms the tip of the T6SS inner tube that is translocated into the prey cell (57). The C-terminal of the protein belongs to the (CDD) BTH_I2691 family effector. This has a predicted structure similar to colicin Ia (58), a bacteriocin that kills gram-negative bacteria by forming a pore in their inner membrane (59). Since antibacterial pore-forming MIX effector proteins are typically encoded upstream of cognate immunity protein(s) that protect the cell from autolysis (60), it is plausible that OG0000002 encodes such immunity proteins. *V. vulnificus* is known to encode two T6SSs; T6SS2 is found in all, while T6SS1 is only in some isolates (61). Both of these have been shown to exert both intra- and interspecies antibacterial activity and are believed to provide the host cell with a competitive advantage (61, 62). Similar to many other MIX effector proteins, the Cluster 4 effector protein is not encoded within the T6SS loci but probably uses one or both of them for its secretion. Interestingly, the promoter region of OG0003626 has a binding site for SmcR (63), a quorum-sensing regulator and homolog of LuxR in *Vibrio fischeri* (64).

Cluster 12 consists of 12 enriched orthologs located on chromosome II (Fig. S5). Six of these are located within the same operon that is present in all clinical isolates. The operon encodes an RTX toxin-like protein (OG0000086; VV2_1514), a putative RTX toxin secretion ATP-binding protein (OG0003730; VV2_1515), a type I secretion membrane fusion protein (OG0003736; VV2_1516), a TolC family outer membrane protein (OG0003735; VV2_1517), an OmpA-like domain-containing protein (OG0003662; VV2_1518), and an ABC-type phosphate transport system protein (OG0003678; VV2_1519). The large (4654 aa) RTX toxin-like protein encodes a Ca+ stabilized adhesin repeat and the conserved FhaB domain, which is present in large exoproteins involved in heme utilization or adhesion (65). Although the protein displays sequence similarity with a putative repeat-in-toxins (RTX) family exoprotein in *E. coli,* it has distinct features from typical RTX proteins (65). Deletions in the gene in *V. vulnificus* YJ016 did not affect cell adherence, cytotoxicity, or virulence. Instead, the gene was shown to display increased expression under iron-limiting conditions (65). Iron availability plays a pivotal role in the pathogenesis of *V. vulnificus* infection and growth (66). To our knowledge, this is the first report linking this ortholog to the pathogenicity of *V. vulnificus*.

Additional details on clusters 2, 5, 10, 13, and 15 are provided in Appendix S1 and Fig. S6 through S9.

*V. vulnificus* is known for its high invasiveness and capacity to damage host cells, partly due to the expression of several exotoxins (48). These include the multifunctional autoprocessing repeats‐in‐toxin (MARTX) toxin, in *V. vulnificus* named RtxA (67), phospholipase A2 (PlpA) (68), cytolysin/hemolysin (VvhA) (69), and elastolytic protease (VvpE) (70). Despite the fact that at least two of these, RtxA and PlpA, have proven to be essential for pathogenicity in *V. vulnificus,* none were identified by our method. The reason is that the orthologs representing these toxins are generally present across all strains, except for RtxA, which is absent in a clade of fourteen Baltic Sea environmental isolates and in one clinical isolate (Fig. S10). This highlights a weakness of the employed method; orthologs not displaying a difference in presence between the two sets of isolates will not be detected, although they may provide the bacteria with pathogenic potential.

### Convergent gene losses of clinical-enriched orthologs indicate a shared ecological niche

Most of the clinically enriched orthologs display one of two distinct occurrence patterns among the *V. vulnificus* strains. Of the 128 orthologs, 72 are found in 91%–100% of the clinical isolates, while being present in 64%–90% of the environmental isolates. Meanwhile, 45 are present in 9%–22% of the clinical isolates and <1%–10% of the environmental isolates (Fig. S11a). Ancestral state reconstruction of the orthologs’ presence/absence revealed that all orthologs in the first category had a high likelihood (>96%) of being present at the root of the tree (Fig. S11b). This suggests these genes were present in the last common ancestor of *V. vulnificus* but subsequently lost in ancestors of certain clades, primarily those including environmental isolates. In contrast, the 45 orthologs with low prevalence had low likelihood (<20%) of being present in the last common ancestor, indicating that they were more likely acquired through multiple independent gene gain events.

Notably, a clade of 19 environmental strains in L2e, along with a clade of four and a clade of one strain in L1, lack a similar set of orthologs that constitute the majority (49–56) of the 72 clinically enriched orthologs that were present in the last common ancestor. This indicates these genes were independently lost in the three clades; hence an example of convergent evolution. The lost genes are mainly part of co-localization clusters 3, 14, and 13 on chromosome 1 and clusters 5, 9, 10, 11, and 12 on chromosome 2 (Fig. 4). Given that these clusters are located at distant loci across both chromosomes, their losses within each clade were unlikely caused by single deletion events, but rather multiple independent deletions, reinforcing the observation of this pattern across different parts of the phylogenetic tree.

Clusters 5, 12, 11, and 10 are located (in this order) in a ~250 kb region on chromosome 2. A possible scenario is that these genes were initially lost in one clade, and subsequently, the whole chromosomal region replaced the corresponding regions in the other two clades through homologous recombination. If this occurred, the remaining genes between the lost co-localization clusters should exhibit a different phylogeny from the backbone genome tree; the 24 strains with the deletions should form a monophyletic group. To test this hypothesis, we constructed a phylogenetic tree on a concatenation of 82 orthologs located in the regions between clusters 5, 12, 11, and 10. While the tree displayed notable differences from the backbone tree, analogous to variations observed in comparisons of phylogenies for the two chromosomes (23), the isolates still clustered within the same major lineages as in the backbone tree, including the 24 isolates carrying the deletions (Fig. S12). Therefore, the chromosomal segment replacement scenario appears unlikely, suggesting instead that a more complex evolutionary process resulted in the parallel deletions. The convergent evolution indicates that selection has favored having either all or none of these gene clusters, which include clusters for CPS formation, biofilm formation, pilus synthesis, and more, implying they are functionally connected, and that having or not having them is linked to distinct ecological niches or strategies that *V. vulnificus* can explore in its natural environment. Since these strains lack essential genes for CPS formation, we can be rather sure they are non-pathogenic. It is also noteworthy that these globally distributed strains constitute more than half of the strains that lack essential genes for CPS. Revealing how the niche and environmental drivers of these likely non-pathogenic strains differ from other *V. vulnificus* strains is relevant for proper risk assessment of *V. vulnificus*.

### Potential biomarkers for monitoring of pathogenic *V. vulnificus*

Since *V. vulnificus* infections can rapidly progress into fatal septicemia in susceptible individuals, assays for fast and accurate detection of *V. vulnificus* in environmental (water, sediment) and seafood samples are desirable. Such an assay should specifically detect *V. vulnificus*. Ideally, it should also be specific to pathogenic strains of the species, but more importantly, not miss any pathogenic strains, i.e., have a high sensitivity. Genotyping approaches based on genetic variation within individual loci, such as the 16S rRNA, *vcg* (VV1_0734), and *pilF* (VV1_2773) genes, have been proposed to distinguish pathogenic from non-pathogenic *V. vulnificus* strains (71–73). However, the discriminatory power of these loci has been challenged (74, 75). Given that the phylogenetic structure is poorly correlated to clinical vs. environmental isolation source (Fig. 2), it seems unlikely (although not impossible) that polymorphism in individual genes would correlate well with pathogenicity. An alternative approach is to base the assay on the presence of a specific gene that occurs in a large fraction of the pathogenic strains of the species while being less frequent in the non-pathogenic ones.

To pinpoint potential biomarkers for identifying pathogenic *V. vulnificus*, we selected a set of “core clinically enriched orthologs” from the pool of the 128 clinically enriched orthologs based on two criteria: the candidates should (i) be present in at least 99.5% of the clinical isolates and (ii) exhibit among the highest correlations with clinical isolation source, as determined by the phylogenetic generalized logistic regression (Fig. S13; Table S4). For seven of these orthologs, we designed primer pairs that were specific to *V. vulnificus* using the degprimer_design and degprimer_specificity pipelines developed as part of this study (Materials and Methods). To assess the efficacy of these primers, we conducted an *in silico* comparative analysis with existing primers for *V. vulnificus* detection (Table S5). Unlike the existing primers, all of our seven candidate primer pairs demonstrated a match rate exceeding 99.5% in clinical isolates, with a notably greater proportion of matches in clinical compared to environmental strains (83–88%; Table S6). The fact that it was impossible to design primers matching nearly all clinical strains while at the same time matching nearly none of the environmental ones is probably reflecting that a substantial fraction of the environmental strains have the genetic potential to be clinical, i.e., are pathogenic. We also designed two primer pairs that target all *V. vulnificus* strains, with 100% match across all strains (Table S6). All of the newly designed primers had at least three mismatches relative to genomes of other *Vibrio* species, as determined by the Degprimer_specificity pipeline. The designed primer pairs were tested in PCR (Fig. S14). Seven of the nine primer pairs gave single-band amplification of DNA from a clinical *V. vulnificus* strain (ATCC 27562), while not giving amplification on DNA from a Baltic Sea water sample sampled in early spring (3°C water temperature) when *V. vulnificus* is expected to be absent. These results support the newly designed primer’s specificity and indicate their potential for surveillance purposes.

In conclusion, this study provides new insights into the genomic basis of virulence and ecological differentiation in *V. vulnificus*. By analyzing 407 genomes with a phylogeny-aware approach, we identified 128 orthologs significantly enriched in clinical isolates. The organization of these genes into co-localization clusters supports the existence of functionally linked pathogenicity modules, including those involved in capsular synthesis, biofilm formation, pilus assembly, and responses to host-like conditions. The observed pattern of convergent gene loss across multiple environmental clades suggests adaptation to a non-pathogenic ecological niche, highlighting the evolutionary plasticity of this species. Identifying core clinically enriched orthologs enabled the design of novel, highly specific PCR primers for potential use in monitoring pathogenic *V. vulnificus*. These findings expand our understanding of virulence evolution in this species and lay the groundwork for improved diagnostic and surveillance strategies.

## MATERIALS AND METHODS

### Sampling, isolation, culturing, and DNA extraction

Following the salinity gradient of the Baltic Sea, samples from 19 stations were collected from July 25th to September 2nd, 2021, covering the German, Estonian, Finnish, Polish, Lithuanian, Swedish, and Danish coasts. From five of these stations (Fig. 1), microbial isolates were selected for subsequent genomic analyses. Sampling, isolation, and identification of *V. vulnificus* were performed as described in Riedinger et al. (15). A detailed description can be found in Appendix S2.

### DNA sequencing and genome assembly

Shotgun libraries for 84 isolates were prepared using Illumina DNA (Flex) and sequenced together on one Illumina NovaSeq6000 run (2 × 150 bp paired-end reads). Ten of the isolates that obtained too little data were resequenced on one MiSeq (2 × 250 bp paired-end-reads). Reads pre-processing (adapter and low-quality bases removal) was carried out with Fastp (params: --cut_mean_quality 25) (76) and genome assembly with Shovill (v1.1.0, default parameters) (77). Assembly completeness and contamination were evaluated using CheckM (78). Two genomes were removed because of strain heterogeneity (>33%), indicating contamination (>83%). The remaining 82 *V. vulnificus* genomes had an estimated completeness of 100% and contamination of 0% and were used for the analyses in this study. We deposited the genomes into GenBank under accession numbers SAMN40604317–SAMN40604398 under the BioProject accession no. PRJNA1091677.

### Comparative genomics with PhyloBOTL

The comparative genomic analysis of *V. vulnificus* encompassed 208 clinical isolates (including 47 sourced from the Baltic Sea region) and 199 environmental isolates (86 from the Baltic Sea, of which 82 were generated in this study), as detailed in Table S2. Environmental isolates included isolates from water, sediment, and sand sources. Isolates from animal sources were not included. The previously published genome sequences were downloaded from NCBI on January 10, 2023. All 407 genomes exhibited estimated completeness >86% (mean 99.92%) and contamination <9.7% (mean 0.17%) (Table S2).

**Fig 5:**
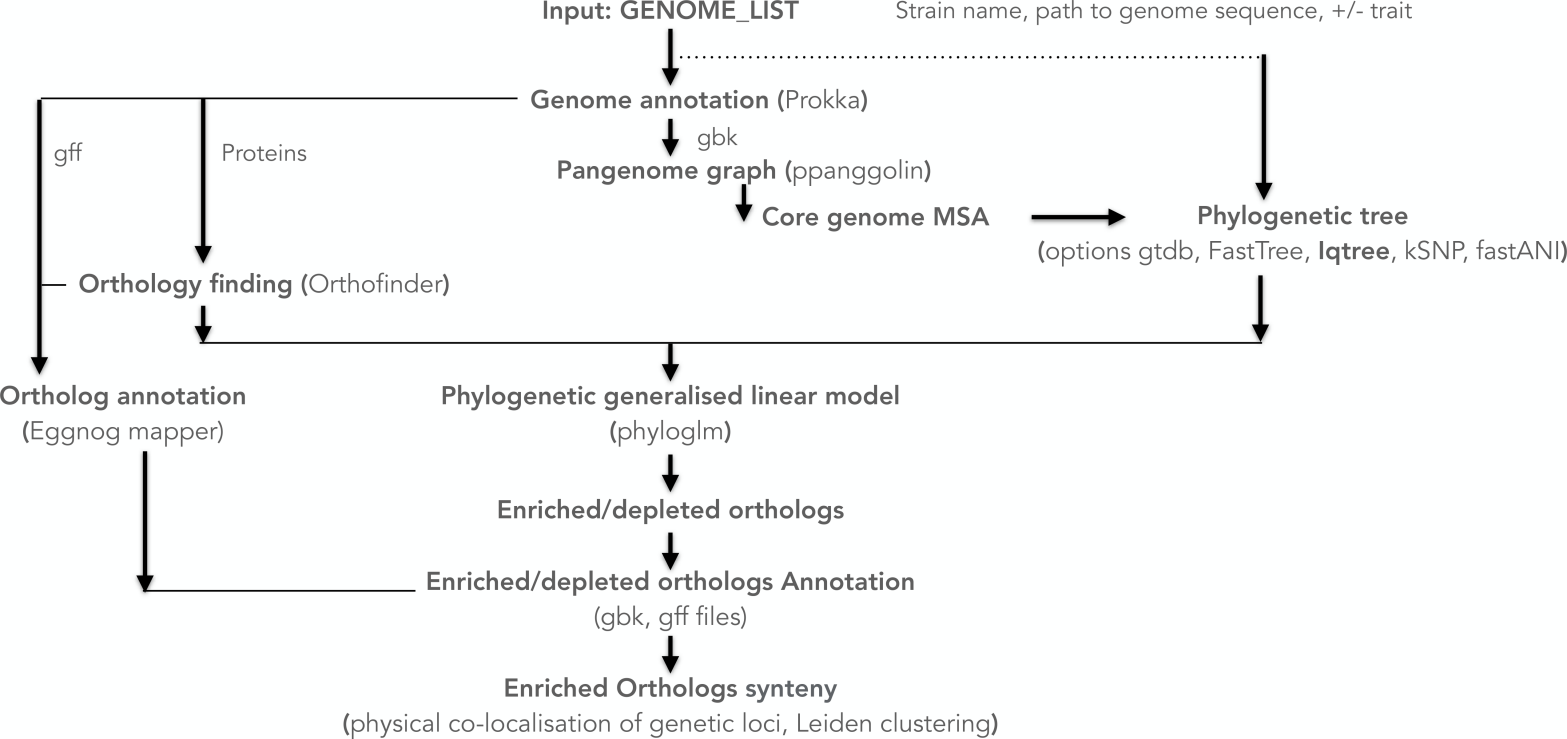
Outline of the PhyloBOTL pipeline workflow.

The comparative genomics analysis was executed with PhyloBOTL (https://github.com/EnvGen/phyloBOTL). PhyloBOTL is a comparative genomics pipeline that, starting with a set of genome sequences for a species, automatically performs gene prediction, ortholog finding and annotation, and phylogenetic reconstruction, using state-of-the-art tools. It further performs gene–trait association analysis by employing a phylogeny-aware statistical model (29) to identify genes (orthologs) whose gain or loss coevolves with a trait over the course of evolution. Several methods exist for each of these steps (e.g., Prokka (79), eggNOG mapper v2 (80), IQ-tree2 (81), PPanGGolin (82), OrthoFinder (83), Roary (84), Scoary (85), Panaroo (86), and Pyseer (87)), but to our knowledge, PhyloBOTL is unique in conducting all steps. Additionally, it performs clustering on the trait-associated orthologs based on the genes’ proximity within the genomes, which can reveal functionally related modules. Finally, it outputs graphs illustrating the genetic organization of each gene cluster within the genomes. The pipeline workflow is illustrated in Fig. 5 and detailed in Appendix S2.

### Primer design and specificity

The process of identifying candidate biomarkers for *V. vulnificus* pathogenicity involved utilizing enriched orthologous DNA sequences. To design primers, we developed and employed the pipeline Degprimer_design, accessible at https://github.com/envgen/Degprimer_design. To evaluate primer specificity, we developed and employed the Degprimer_specificity pipeline, accessible at https://github.com/envgen/Degprimer_specificity. A detailed description of the Degprimer_design and Degprimer_specificity pipelines can be found in Appendix S2.

### PCR evaluation of primers

The nine designed primer pairs were evaluated in PCRs on *V. vulnificus* ATCC 27562 DNA (DSMZ, Braunschweig, Germany), as well as on DNA from a Baltic Sea water sample from early spring, where *V. vulnificus* was expected to be absent. ATCC 27562 encodes all of the clinically enriched orthologs that the primers were designed to target. Gradient PCR identified annealing temperatures (Table S6) for seven out of the nine primers, where DNA from *V. vulnificus* generated a single band, while DNA from the water sample gave no amplification. See Appendix S2 for details.

## Data Availability

The genome assemblies are available at the NCBI Assembly database (https://www.ncbi.nlm.nih.gov/assembly/) under BioSample accession numbers SAMN40604317–SAMN40604398, corresponding to the BioProject accession number PRJNA1091677. The following supplemental data sets are available on Zenodo at https://doi.org/10.5281/zenodo.16754641: Data set 1, phylogenetic tree in Newick format of the 407 *V. vulnificus* genomes; Data set 2, counts of the OGs in the 407 *V. vulnificus genomes*; Data set 3, protein sequences in fasta format for all 13,920 OGs, one representative sequence per OG; Data set 4, gene (DNA) sequences in fasta format for all 13,920 OGs, one representative sequence per OG. The data analysis of short DNA sequencing reads was performed using open-source software tools, as detailed in Materials and Methods. For comparative analysis, we developed and employed the PhyloBOTL pipeline (https://github.com/EnvGen/phyloBOTL). We also developed the Degprimer_design pipeline for primer design (https://github.com/envgen/Degprimer_design) and the Degprimer_specificity pipeline for evaluating primer specificity (https://github.com/envgen/Degprimer_specificity).

## SUPPLEMENTAL MATERIAL

Appendix S1 mbio.00205-26-s0001.pdf Description of Clusters 2, 5, 10, 13, and 15.

Appendix S2 mbio.00205-26-s0002.pdf Supplemental methods.

File S1 mbio.00205-26-s0003.html Interactive map of sampling locations, including substations, for the *Vibrio vulnificus* isolates.

Supplemental figures mbio.00205-26-s0004.pdf Figures S10 to S14.

Legend mbio.00205-26-s0005.docx File S1 legend.

Fig. S1 to S3 mbio.00205-26-s0006.pdf Presence and top 5 most common gene arrangements within co-localization Clusters 3, 11, and 14.

Fig. S4 to S6 mbio.00205-26-s0007.pdf Presence and top 5 most common gene arrangements within co-localization Clusters 4, 12, and 5.

Fig. S7 to S9 mbio.00205-26-s0008.pdf Presence and top 5 most common gene arrangements within co-localization Clusters 10, 15, and 13.

Supplemental tables mbio.00205-26-s0009.xlsx Tables S1 to S6.

ASM does not own the copyrights to Supplemental Material that may be linked to, or accessed through, an article. The authors have granted ASM a non-exclusive, world-wide license to publish the Supplemental Material files. Please contact the corresponding author directly for reuse.
