# Supplementary material for: Phylogeny-aware comparative genomics of Vibrio vulnificus links genetic traits to pathogenicity
Source: mBio. 2026 Jun 17;17(7):e00205-26. doi: 10.1128/mbio.00205-26 (PMC13348674; doi:10.1128/mbio.00205-26)
Supplement: Appendix S1 — Description of Clusters 2, 5, 10, 13, and 15. [file mbio.00205-26-s0001.pdf]

Supplementary Appendix 1: Description of Clusters 2, 5, 10, 13 and 15

Cluster 2 includes six enriched orthologues on chromosome I, all with unknown functions except OG0003857 annotated as Catechol 2,3-dioxygenase-like lactoylglutathione lyase family enzyme and with OG0003707 (VV1_2557) annotated as putative exported protein (Fig. S6). The ortholog OG0003785 (VV1_2555) was shown to be upregulated in *V. vulnificus* CMCP6 under elevated c-di-GMP conditions (1).

Cluster 5 comprises three enriched orthologues on chromosome 2 (Fig. S7), OG0004016 (VV2_1354) a transcriptional regulator, OG0003703 (VV2_1396), a transcriptional regulator with a LuxR-type DNA-binding HTH domain, and OG0003664 (VV2_1397), encoding a MSCRAMM family adhesin clumping factor ClfA region. MSCRAMM ("microbial surface components recognizing adhesive matrix molecules") adhesin proteins have mainly been described in gram-positive bacteria, where they mediate attachment to host tissue. Known targets are fibrinogen and fibronectin (2),(3). Clumping factor A (ClfA) is an important virulence factor of *Staphylococcus aureus* that binds to the blood plasma protein fibrinogen (4)

Cluster 10 encodes potential spermidine synthases. Spermidine has been shown to protect *Salmonella enterica* against ROS-mediated cytotoxicity (5) and spermidine transporters are upregulated in *V. vulnificus* grown in serum (6). It includes three enriched orthologues: OG0003691 (VV2_1635) and OG0003690 (VV2_1636) are organized in the same operon and encode a spermidine synthase and a fused Major Facilitator Superfamily (MFS)/spermidine synthase (Fig. S8), respectively. Additionally, OG0003634 (VV2_1626) is located in a second operon and is classified as an acyltransferase, as indicated in the Virulence Factor Database (VFDB), where it is linked to immune modulation (VFG048946). Polyamines, including putrescine, cadaverine, spermidine, and spermine, are low molecular weight aliphatic hydrocarbons with quaternary nitrogen groups, carrying a net positive charge under physiological pH conditions. They play a crucial role in maintaining the optimal conformation of nucleic acids, thereby being indispensable for normal cellular growth and proliferation (7). In bacteria, polyamines serve diverse functions, including scavenging free radicals to confer acid resistance, promoting biofilm formation, and regulating the expression of virulence factors. They also mediate immune evasion during bacterial infection establishment (7–10). Due to their positively charged and flexible backbone, polyamines are frequently integrated into natural products, particularly iron-scavenging siderophores (9). This is particularly relevant to *V. vulnificus*, which utilizes siderophore-based systems to sequester iron from high-affinity binding proteins like ferritin, transferrin, and lactoferrin (6, 11, 12). Williams et al. (2014) (6) utilized RNA sequencing to examine the transcriptome of *Vibrio vulnificus* C-genotypes following exposure to human

serum compared to seawater. Their findings revealed an upregulation of spermidine transport genes (potABCD operon) in human serum, indicating a potential role for polyamines, specifically spermidine, in the physiological functions of *V. vulnificus* within the human host. Consistent with these results, our investigation revealed a significant enrichment of genes associated with spermidine synthase in clinical isolates. However, to date, there is no published study evaluating the impact of spermidine on *Vibrio vulnificus* pathogenicity, warranting further investigations.

Cluster 13 includes three enriched orthologues on chromosome I: OG0003605 (VV1_3127), a diguanylate cyclase response regulator, OG0003625 (VV1_3128), a signal transduction histidine kinase response regulator in one operon, and OG0003632 (VV1_3142) (Fig. S9). OG0003632 is classified as a methyl-accepting chemotaxis protein (COG0840) and comprises a Cache 3/Cache 2 fusion domain (PF17201) that serves as an extracellular sensor, binding to environmental signaling molecules (13), such as nutrients. Only a small number of Cache domains have known ligands, and the ligand to the gene product of OG0003632 in *V. vulnificus* is, to our knowledge, unknown.

Cluster 15 includes 10 enriched orthologues located on chromosome I. Four of them are components of the *ttrRSBCA* locus for tetrathionate respiration (14); three constitute the *ttrBCA* operon (OG0004780-OG0004811-OG0004812) that encodes the subunits of the tetrathionate reductase complex, and OG0004813 encodes *ttrS*, a sensory histidine kinase involved in the *ttr* regulation. The *ttr* operon enables the utilization of tetrathionate as an electron acceptor, thus facilitating respiration in anaerobic environments such as in sediments or inside the mammalian gut (15). This operon is a potent virulence factor for several enteric pathogens, most prominently *Salmonella enterica* (16). This cluster is however only present in some *V. vulnificus* isolates (S10 Fig).

A common theme among several co-localisation clusters. Several of the co-localisation clusters are associated with c-di-GMP. This molecule plays a key role in controlling the transition from a motile to a sessile lifestyle. Like several other pathogenic bacteria, *V. vulnificus* can exist in a free-living planktonic state or a surface-attached biofilm state (17). Biofilms provide resistance to environmental stressors, and in the case of *V. vulnificus*, a means for colonization and accumulation in shellfish. Biofilm formation is triggered by external stimuli, for example high calcium levels, via c-di–GMP (18). At least two of the enriched clusters (11 and S16) encode genes related to biofilm formation. Once biofilms have grown dense and matured, quorum-sensing can trigger the cells to detach and transition to a planktonic state, leading to dispersal of the cells and potentially tissue colonization when inside the human body. This is governed by the master regulator SmcR, a homolog of LuxR, that senses the cell density with the extracellular concentration of the autoinducer-2 (AI-2) via a signal transduction cascade (19). SmcR triggers CPS

formation (Cluster 2) and the CPS inhibits further biofilm formation and protects the cells in their planktonic state. When inside the mammalian body, CPS plays a critical role in evading the host's innate immune system by providing antiphagocytic ability and resistance to complement-mediated killing (20). SmcR expression can also be triggered by mammalian host cells by activating LuxS, an autoinducer-2 synthase (19). In addition to the CPS and biofilm clusters, genes related to c-di-GMP, quorum-sensing and SmcR were found in several clusters: Clusters 2, 11 and 14 include orthologs earlier shown to be differentially expressed at elevated c-di-GMP levels (1), Cluster 4 and 13 encode diguanylate cyclases that produce c-di-GMP in response to specific environmental signals, Cluster 15 encodes an EAL-domain phosphodiesterase involved in degradation of c-di-GMP, and finally, Cluster 4 includes an ortholog with an SmcR binding site in its promoter region (21), implying its regulation by SmcR.

References

1. Chodur DM, Rowe-Magnus DA. 2018. Complex Control of a Genomic Island Governing Biofilm and Rugose Colony Development in Vibrio vulnificus. J Bacteriol 200.

2. Ganesh VK, Rivera JJ, Smeds E, Ko Y-P, Bowden MG, Wann ER, Gurusiddappa S, Fitzgerald JR, Höök M. 2008. A structural model of the Staphylococcus aureus ClfA-fibrinogen interaction opens new avenues for the design of anti-staphylococcal therapeutics. PLoS Pathog 4:e1000226.

3. Deivanayagam CCS, Wann ER, Chen W, Carson M, Rajashankar KR, Höök M, Narayana SVL. 2002. A novel variant of the immunoglobulin fold in surface adhesins of Staphylococcus aureus: crystal structure of the fibrinogen-binding MSCRAMM, clumping factor A. EMBO J 21:6660–6672.

4. McAdow M, Kim HK, Dedent AC, Hendrickx APA, Schneewind O, Missiakas DM. 2011. Preventing Staphylococcus aureus sepsis through the inhibition of its agglutination in blood. PLoS Pathog 7:e1002307.

5. Nair AV, Singh A, Rajmani RS, Chakravortty D. 2024. Salmonella Typhimurium employs spermidine to exert protection against ROS-mediated cytotoxicity and rewires host polyamine metabolism to ameliorate its survival in macrophages. Redox Biol 72:103151.

6. Williams TC, Blackman ER, Morrison SS, Gibas CJ, Oliver JD. 2014. Transcriptome sequencing reveals the virulence and environmental genetic programs of Vibrio vulnificus exposed to host and estuarine conditions. PLoS One 9:e114376.

7. Shah P, Swiatlo E. 2008. A multifaceted role for polyamines in bacterial pathogens. Mol Microbiol 68:4–16.

8. Rai AN, Thornton JA, Stokes J, Sunesara I, Swiatlo E, Nanduri B. 2016. Polyamine transporter in Streptococcus pneumoniae is essential for evading early innate immune responses in pneumococcal pneumonia. Sci Rep 6:26964.

9. Michael AJ. 2018. Polyamine function in archaea and bacteria. J Biol Chem 293:18693–18701.

10. Banerji R, Kanojiya P, Patil A, Saroj SD. 2021. Polyamines in the virulence of bacterial pathogens of respiratory tract. Mol Oral Microbiol 36:1–11.

11. Andrus CR, Walter M, Crosa JH, Payne SM. 1983. Synthesis of siderophores by pathogenicVibrio species. Curr Microbiol 9:209–214.

12. Payne Shelley M., Mey Alexandra R., Wyckoff Elizabeth E. 2015. Vibrio Iron Transport: Evolutionary Adaptation to Life in Multiple Environments. Microbiol Mol Biol Rev 80:69–90.

13. Upadhyay AA, Fleetwood AD, Adebali O, Finn RD, Zhulin IB. 2016. Cache Domains That are Homologous to, but Different from PAS Domains Comprise the Largest Superfamily of Extracellular Sensors in Prokaryotes. PLoS Comput Biol 12:e1004862.

14. Hensel M, Hinsley AP, Nikolaus T, Sawers G, Berks BC. 1999. The genetic basis of tetrathionate respiration in Salmonella typhimurium. Mol Microbiol 32:275–287.

15. Adsit FG Jr, Randall TA, Locklear J, Kurtz DM. 2022. The emergence of the tetrathionate reductase operon in the Escherichia coli/Shigella pan-genome. Microbiologyopen 11:e1333.

16. Winter SE, Thiennimitr P, Winter MG, Butler BP, Huseby DL, Crawford RW, Russell JM, Bevins CL, Adams LG, Tsolis RM, Roth JR, Bäumler AJ. 2010. Gut inflammation provides a respiratory electron acceptor for Salmonella. Nature 467:426–429.

17. Ashrafudoulla M, Mizan MFR, Park SH, Ha S-D. 2021. Current and future perspectives for controlling Vibrio biofilms in the seafood industry: a comprehensive review. Crit Rev Food Sci Nutr 61:1827–1851.

18. Chodur DM, Coulter P, Isaacs J, Pu M, Fernandez N, Waters CM, Rowe-Magnus DA. 2018. Environmental Calcium Initiates a Feed-Forward Signaling Circuit That Regulates Biofilm Formation and Rugosity in Vibrio vulnificus. MBio 9.

19. Kim SM, Park JH, Lee HS, Kim WB, Ryu JM, Han HJ, Choi SH. 2013. LuxR homologue SmcR is essential for Vibrio vulnificus pathogenesis and biofilm detachment, and its expression is induced by host cells. Infect Immun 81:3721–3730.

20. Pettis GS, Mukerji AS. 2020. Structure, Function, and Regulation of the Essential Virulence Factor Capsular Polysaccharide of Vibrio vulnificus. Int J Mol Sci 21:3259.

21. Lee DH, Jeong HS, Jeong HG, Kim KM, Kim H, Choi SH. 2008. A consensus sequence for binding of SmcR, a Vibrio vulnificus LuxR homologue, and genome-wide identification of the SmcR regulon. J Biol Chem 283:23610–23618.
