# Supplementary material for: Phylogeny-aware comparative genomics of Vibrio vulnificus links genetic traits to pathogenicity
Source: mBio. 2026 Jun 17;17(7):e00205-26. doi: 10.1128/mbio.00205-26 (PMC13348674; doi:10.1128/mbio.00205-26)
Supplement: Appendix S2 — Supplemental methods. [file mbio.00205-26-s0002.pdf]

Supplementary Appendix 2: Supplementary methods

Sampling, isolation, culturing, DNA extraction. Water and sediment samples were collected from within *Zostera marina* meadows (substation A), as well as from the leaves of *Z. marina* plants. Similarly, water and sediment samples were taken at control stations lacking *Z. marina*, located at distances of 15 m (substation B) and 100 m (substation C) from the meadows' edges (see interactive Supplementary File 1). Sampling was conducted by SCUBA or snorkel divers at water depths ranging from 0.6 to 4.7 m.

Vibrio spp. colony forming units (CFUs) and *V. vulnificus* isolates were obtained from water, sediment, and *Z. marina* across six independent replicates. For this, water samples of 50, 100, or 200 µL were (a) directly plated onto Vibrio-selective thiosulfate citrate bile sucrose (TCBS) agar (Merck, Darmstadt, Germany), and (b) aliquots of 2, 5, 10, or 25 mL were filtered onto 0.2 µm PC-filters (Merck-Millipore, Burlington, USA) and placed onto TCBS agar. The sediment samples were homogenized after removing the overlying water; a subsample of approximately 10 g (dry weight determined accurately after lyophilization) was transferred from six sediment samples to sterile 50 mL Falcon tubes, where 40 mL of double 0.2 µm-sterile filtered station water was added. To detach bacteria, five ultrasonic pulses of 10 s at 25% capacity at 5 second intervals using the Bandelin SONOPULS HD 2200.2 (Bandelin, Berlin, Germany) were applied. After subsequent vortexing and settling of the sediment, water samples of 50, 100, or 200 µL were plated on TCBS agar in six biological replicates. For the cultivation of seagrass-associated *Vibrio spp.*, the overlying water was removed; double sterile filtered station water (40 mL) was added to the seagrass samples. To detach bacteria, five ultrasonic pulses of 10s at 25% of capacity at 5-second intervals using the Bandelin SONOPULS HD 2200.2 (Bandelin, Berlin, Germany) were applied to the seagrass samples. After subsequent vortexing and settling of the sediment, between 5 and 20 mL of supernatant was filtered over a PC filter and placed on TCBS-agar in six biological replicates. After 24 h of incubation at 37 °C, CFUs of green colonies were determined for all plates.

To isolate *V. vulnificus*, green colonies were further cultivated on CHROMagar_vibrio™ (Chromagar Ltd. Paris, France) for 24 h at 37 °C, and blue-colored colonies were restreaked on TCBS agar (Carl Roth, Karlsruhe, Germany), CHROMagar (Mast Diagnostica, Reinfeld, Germany), and Columbia sheep blood agar (Oxoid, Basingstoke, UK). DNA of isolates that were green on TCBS, blue on CHROMagar, and confirmed to be pure cultures on blood agar was extracted (DNeasy Blood and Tissue Kit, Qiagen, Hilden, Germany), and purity and concentration were determined using a NanoDrop Spectrophotometer (Thermo Fisher, Waltham, USA). To confirm *V. vulnificus*, the species-specific *vvhA* gene sequence was targeted using multiplex real-time PCR (5′ nuclease assay). In the same assay, an internal amplification control

(KOMA) was analysed. Primers and probes for *vvhA* detection were used as described by Messelhäusser et al. (2010) (1).

Comparative genomics with PhyloBOTL. PhyloBOTL comprises the following steps (with the parameter settings used in this study indicated):

1. Annotation of genome sequences with Prokka (v1.14.6 with default parameters) (2).

2. Multiple sequence alignment (MSA) of the coding core-genome with ppanggolin (v1.2.105, module ppanggolin msa) (3).

3. Phylogenetic tree inference based on the MSA with maximum likelihood, utilizing IQ-TREE (v2.2.3, parameters: -m GTR+I+G -B 1000 -bnni; and -m 12.12 --seqtype DNA for tree rooting) (4).

4. Identification of orthologous groups of genes with Orthofinder (v2.5.5, parameters: -S diamond -og) (5).

5. Association of orthologs in the genomes to traits of the organisms (in this case, clinical versus environmental source of isolation). We employed the Phylogenetic Generalized Linear Model, utilizing implemented in the R package phylolm (6), which is grounded in an evolutionary model for binary traits, where trait values shift between 0 (environmental isolate) and 1 (clinical isolate) as species evolve along the phylogenetic tree (7). The R package logistf (8), which implements Firth's Bias-Reduced Logistic Regression, was employed to achieve precise coefficient estimations when the phylogenetic signal was negligible (alpha > 1628). Firth's method is recognized as an effective solution to the separation issue in logistic regression (9). The alpha threshold is defined by the equation log(alpha * T) = 4 (7), where 4 represents the upper limit (6) and T denotes the mean tree tip height. In our analysis, we established the threshold at exp(4) * 0.95 / T, corresponding to 95% of the upper limit values of alpha, which in our case amounts to 1628.

6. Functional annotation of orthologs that are significantly (False Discovery Rate [FDR] adjusted *P*-value < 0.05) enriched or depleted between the traits, using 1) Eggnog mapper (v2.1.12) (10) and the EggNOG database (v5) (11), and 2) mmseqs easy-search (v13.45111, with parameters --max-accept 1 --start-sens 4 --sens-steps 3 -s 7 -v 1 --min-seq-id 0.9) (12) using Uniref90 (13) proteins with a specific taxonomy affiliation (here, *Vibrionacea*) as reference database.

7. Identification of plasmids and prophages in the genomes using genomad (v1.6.1, with flags --cleanup --conservative) (14).

8. Grouping the trait-associated orthologs based on proximity in the genomes. Pairs of trait-associated orthologs present within 40 kb distance in at least four genomes were included in the input table to build a network graph, where nodes represent orthologs and edges represent co-occurence in the genomes. The weight of an edge represents the number of genomes in which the ortholog pair co-occurs (within 40 kbp distance). Subsequently, the Leiden graph clustering algorithm, implemented in the R package igraph (15), was applied

to obtain the co-localisation clusters. The visualization of the clusters was performed using the R packages gggenomes (16) and ggtree (17), using the script synteny_visual.R of the phyloBOLT pipeline.

Additional bioinformatic analysis. Outside the PhyloBOTL pipeline, we used AMRFinderPlus (version 3.11.26, database version 2023-09-26.1, using the flag --plus) (18) to detect antimicrobial resistance genes in the *V. vulnificus* genomes. We used operon mapper (19) for operon identification in the YJ016, CMCP6 and MO6-24/O complete genomes, and further mapped the genes *de novo* predicted by our pipeline in these three genomes to the corresponding genes in NCBI with Blastx (20), to obtain the original gene names (provided in Table 2). In addition to the automatic gene annotations, we conducted manual annotations of selected clinically enriched orthologs using NCBI BLAST (20) and CDD-search (21), and by literature searches. The tree in Figure 3 was plotted with function plot.phylo in the R package Ape (22). Ancestral state reconstruction of the orthologs' presence/absence on the phylogenetic tree was performed with function ace in Ape, using default settings (type = 'discrete'). For constructing the phylogenetic tree based only on DNA sequences located between the colocalization clusters 5, 10, 11, and 12, the following was performed: On the FORC_009 genome (a complete genome where the gene clusters are present), we identified all orthologs located in the three regions between the clusters (spanning position 294,475 to 531,541 on chromosome 2). Among these orthologs, we selected those that were 1) present in >90% of all strains, 2) never present in >1 copy per genome. The gene sequences of the 82 selected orthologs from each genome (minus potential missing ones) were subsequently concatenated, in the same order.

Primer design and specificity. The process of identifying candidate biomarkers for *V. vulnificus* pathogenicity involved utilizing enriched orthologous DNA sequences. To design primers, we developed and employed the pipeline Degprimer_design, accessible at https://github.com/envgen/Degprimer_design. This pipeline integrates several programs, including Snakemake v3.13.3 (23), Muscle v3.8.1551 (24), Degeprime v1.1.0 (25) and Mfeprimer v3.2.3 (26). Degprimer_design includes the following steps: 1) generation of a multiple sequence alignment of selected orthologous group sequences using Muscle; 2) alignment trimming using TrimAlignment.pl from Degeprime; 3) degenerate primer design using DegePrime.pl from Degeprime for each primer size and degeneracy defined by the user; 4) screening of primers with GC content and coverage above user-defined thresholds; 5) hairpin prediction of screened primers using Mfeprimer; 6) removal of primers forming hairpins; 7) primer-dimer prediction with Mfeprimer and removal of primers forming dimers. 8) Finally, a list of primer pairs is selected based on the user-defined amplicon size, and melting temperature difference between primer pairs below 5°C.

To evaluate primer specificity, we developed and employed the Degprimer_specificity pipeline, accessible at https://github.com/envgen/Degprimer_specificity. This pipeline incorporates Snakemake 3.13.3 (23), Blastn v2.5.0 (20) and Krona v2.7.1 (27). The Degprimer_specificity pipeline analyzes each primer pair of

a list of primer pairs provided by the user. A Blast search is performed against a user-defined database for each non-degenerate version of each degenerate primer. Results are filtered by selecting primer pairs that fulfill the user-defined cutoffs on percent identity and query coverage, and that generate amplicons in the user-defined size range. *In silico* amplicons are generated and printed to file, together with a Krona file showing the amplicons taxonomic distribution. The output includes a log file (Summary.txt) containing key information, such as the total number of amplicons generated from the target taxon (in this case, *V. vulnificus*) with average amplicon size, as well as the total number of amplicons that are not from the selected taxon. These values can be compared with the values in the Spp_and_strains.txt file, which includes a list and number of species/strains present in the reference database used to check the primer specificity.

Degprimer_specifivity was run on a comprehensive database comprising complete *Vibrio* genomes (51 species, 317 genomes) sourced from The Reference Sequence (RefSeq) project at the National Center for Biotechnology Information (NCBI) (28), alongside 386 draft *V. vulnificus* genomes. This *V. vulnificus* collection includes 43 clinical isolates (29, 30) and 82 environmental isolates from the Baltic Sea, as part of the current study. Moreover, we conducted selectivity evaluations on bacteria using a database of complete genomes from Bacteria Refseq (28), encompassing a comprehensive representation of 1097 genera, 2898 species, and 2931 strains, with a maximum amplicon size of 900 base pairs (no mismatch allowed). For selected primers pairs, primer specificity was further evaluated using the Degprimer_specificity pipeline, allowing up to three mismatches against a dataset of 77 representative *Vibrio* complete genomes from NCBI (74 species, 77 strains, including one *Vibrio vulnificus* strain), with a maximum amplicon size of 900 base pairs.

Evaluation of primers in PCR. Gradient PCRs were conducted on the nine designed primer pairs with annealing temperatures extending from -2 to +2 °C relative to the primers' calculated melting temperatures (Tm) on either *V. vulnificus* ATCC 27562 DNA or DNA from a Baltic Sea water sample. The sea water sample was collected from the surface at an off-shore station in the Baltic proper (56.116333°, 16.530667°) in March (2023-03-09), when the water temperature was 3°C. Hence, *V. vulnificus* was not expected to be present. The PCR products were assessed on agarose gel. For seven of the primer pairs, a temperature was found (Supplementary Table 6) that generated a *V. vulnificus* PCR product of the expected size while not giving background amplification on the sea water DNA. The PCR products (positive and negative) from the optimal Tms for the seven successful primer pairs were subsequently run on a Tapestation (Agilent Technologies, Waldbronn, Germany) and the resulting graphs presented in Supplementary Figure 14. PCRs were run in 20 µl reactions with 1 x KAPA HiFi HotStart ReadyMix (Roche, Basel, Switzerland), 0.5 µM concentration of each primer (Integrated DNA Technologies, Leuven, Belgium), and either 0.2 ng DNA

from *V. vulnificus* or 2.5 ng from sea water. The PCR conditions were 95°C for 3 min, 30 cycles of 98°C for 20 s, [varying]°C for 15 s and 72°C for 15 s, followed by a final elongation step of 72°C for 15 s.

References

1. Messelhäusser U, Colditz J, Thärigen D, Kleih W, Höller C, Busch U. 2010. Detection and differentiation of Vibrio spp. in seafood and fish samples with cultural and molecular methods. Int J Food Microbiol 142:360–364.

2. Seemann T. 2014. Prokka: rapid prokaryotic genome annotation. Bioinformatics 30:2068–2069.

3. Gautreau G, Bazin A, Gachet M, Planel R, Burlot L, Dubois M, Perrin A, Médigue C, Calteau A, Cruveiller S, Matias C, Ambroise C, Rocha EPC, Vallenet D. 2021. Correction: PPanGGOLiN: Depicting microbial diversity via a partitioned pangenome graph. PLoS Comput Biol 17:e1009687.

4. Minh BQ, Schmidt HA, Chernomor O, Schrempf D, Woodhams MD, von Haeseler A, Lanfear R. 2020. Corrigendum to: IQ-TREE 2: New Models and Efficient Methods for Phylogenetic Inference in the Genomic Era. Mol Biol Evol 37:2461.

5. Emms DM, Kelly S. 2019. OrthoFinder: phylogenetic orthology inference for comparative genomics. Genome Biol 20:238.

6. Ho L si T, Ané C. 2014. A linear-time algorithm for Gaussian and non-Gaussian trait evolution models. Syst Biol 63:397–408.

7. Ives AR, Garland T Jr. 2010. Phylogenetic logistic regression for binary dependent variables. Syst Biol 59:9–26.

8. Heinze G, Ploner M, Jiricka L, Steiner G. logistf: Firth's Bias-Reduced Logistic Regression. R package version 1.26.0.

9. Heinze G, Schemper M. 2002. A solution to the problem of separation in logistic regression. Stat Med 21:2409–2419.

10. Cantalapiedra CP, Hernández-Plaza A, Letunic I, Bork P, Huerta-Cepas J. 2021. eggNOG-mapper v2: Functional Annotation, Orthology Assignments, and Domain Prediction at the Metagenomic Scale. Mol Biol Evol 38:5825–5829.

11. Huerta-Cepas J, Szklarczyk D, Heller D, Hernández-Plaza A, Forslund SK, Cook H, Mende DR, Letunic I, Rattei T, Jensen LJ, von Mering C, Bork P. 2019. eggNOG 5.0: a hierarchical, functionally and phylogenetically annotated orthology resource based on 5090 organisms and 2502 viruses. Nucleic Acids Res 47:D309–D314.

12. Hauser M, Steinegger M, Söding J. 2016. MMseqs software suite for fast and deep clustering and searching of large protein sequence sets. Bioinformatics 32:1323–1330.

13. Suzek BE, Huang H, McGarvey P, Mazumder R, Wu CH. 2007. UniRef: comprehensive and non-redundant UniProt reference clusters. Bioinformatics 23:1282–1288.

14. 2023. Fast and accurate identification of plasmids and viruses in sequencing data using geNomad. Nat Biotechnol https://doi.org/10.1038/s41587-023-01982-7.

15. Csárdi G, Horvát S, Müller K, Nepusz T, Noom D, Salmon M, Traag V, Zanini F. 2019. igraph: network analysis and visualization. R package version 1.2. 4.1.

16. Hackl T, Ankenbrand MJ. gggenomes: a grammar of graphics for comparative genomics. R package version 09.

17. Yu G. 2022. Data Integration, Manipulation and Visualization of Phylogenetic Trees. CRC Press.

18. Feldgarden M, Brover V, Gonzalez-Escalona N, Frye JG, Haendiges J, Haft DH, Hoffmann M, Pettengill JB, Prasad AB, Tillman GE, Tyson GH, Klimke W. 2021. AMRFinderPlus and the

Reference Gene Catalog facilitate examination of the genomic links among antimicrobial resistance, stress response, and virulence. Sci Rep 11:12728.

19. Taboada B, Estrada K, Ciria R, Merino E. 2018. Operon-mapper: a web server for precise operon identification in bacterial and archaeal genomes. Bioinformatics 34:4118–4120.

20. Camacho C, Coulouris G, Avagyan V, Ma N, Papadopoulos J, Bealer K, Madden TL. 2009. BLAST+: architecture and applications. BMC Bioinformatics 10:421.

21. Wang J, Chitsaz F, Derbyshire MK, Gonzales NR, Gwadz M, Lu S, Marchler GH, Song JS, Thanki N, Yamashita RA, Yang M, Zhang D, Zheng C, Lanczycki CJ, Marchler-Bauer A. 2023. The conserved domain database in 2023. Nucleic Acids Res 51:D384–D388.

22. Paradis E, Schliep K. 2019. ape 5.0: an environment for modern phylogenetics and evolutionary analyses in R. Bioinformatics 35:526–528.

23. Köster J, Rahmann S. 2012. Snakemake—a scalable bioinformatics workflow engine. Bioinformatics.

24. Edgar RC. 2004. MUSCLE: multiple sequence alignment with high accuracy and high throughput. Nucleic Acids Res 32:1792–1797.

25. Hugerth LW, Wefer HA, Lundin S, Jakobsson HE, Lindberg M, Rodin S, Engstrand L, Andersson AF. 2014. DegePrime, a program for degenerate primer design for broad-taxonomic-range PCR in microbial ecology studies. Appl Environ Microbiol 80:5116–5123.

26. Wang K, Li H, Xu Y, Shao Q, Yi J, Wang R, Cai W, Hang X, Zhang C, Cai H, Qu W. 2019. MFEprimer-3.0: quality control for PCR primers. Nucleic Acids Res 47:W610–W613.

27. Ondov BD, Bergman NH, Phillippy AM. 2011. Interactive metagenomic visualization in a Web browser. BMC Bioinformatics 12:385.

28. Haft DH, DiCuccio M, Badretdin A, Brover V, Chetvernin V, O'Neill K, Li W, Chitsaz F, Derbyshire MK, Gonzales NR, Gwadz M, Lu F, Marchler GH, Song JS, Thanki N, Yamashita RA, Zheng C, Thibaud-Nissen F, Geer LY, Marchler-Bauer A, Pruitt KD. 2018. RefSeq: an update on prokaryotic genome annotation and curation. Nucleic Acids Res 46:D851–D860.

29. Brehm TT, Berneking L, Sena Martins M, Dupke S, Jacob D, Drechsel O, Bohnert J, Becker K, Kramer A, Christner M, Aepfelbacher M, Schmiedel S, Rohde H, German Vibrio Study Group. 2021. Heatwave-associated Vibrio infections in Germany, 2018 and 2019. Euro Surveill 26.

30. Amato E, Riess M, Thomas-Lopez D, Linkevicius M, Pitkänen T, Wołkowicz T, Rjabinina J, Jernberg C, Hjertqvist M, MacDonald E, Antony-Samy JK, Dalsgaard Bjerre K, Salmenlinna S, Fuursted K, Hansen A, Naseer U. 2022. Epidemiological and microbiological investigation of a large increase in vibriosis, northern Europe, 2018. Euro Surveill 27.
