# Supplementary material for: Phylogeny-aware comparative genomics of Vibrio vulnificus links genetic traits to pathogenicity
Source: mBio. 2026 Jun 17;17(7):e00205-26. doi: 10.1128/mbio.00205-26 (PMC13348674; doi:10.1128/mbio.00205-26)
Supplement: Supplemental figures — Figures S10 to S14. [file mbio.00205-26-s0004.pdf]

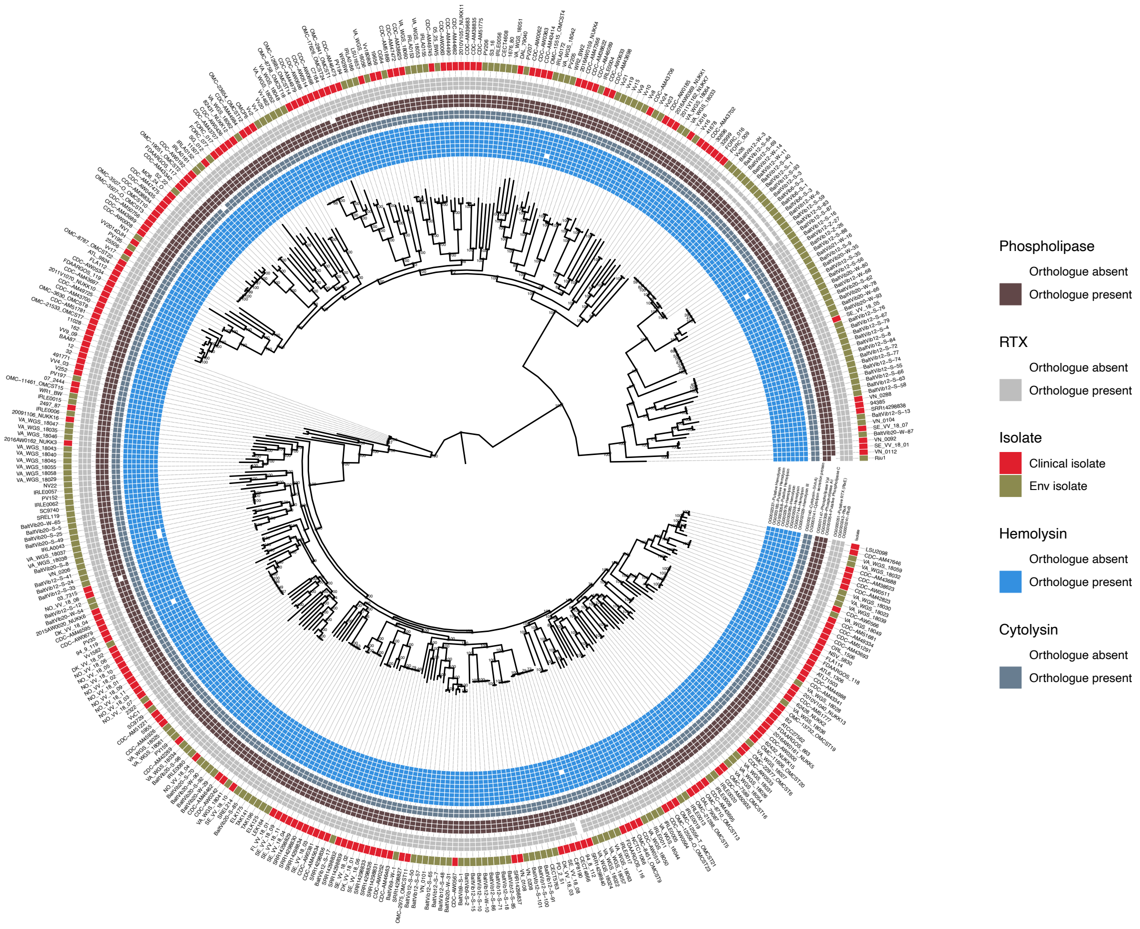

Supplementary Figure 10. Presence of orthologs related to *V. vulnificus* toxins in the 407 *V. vulnificus* genomes.

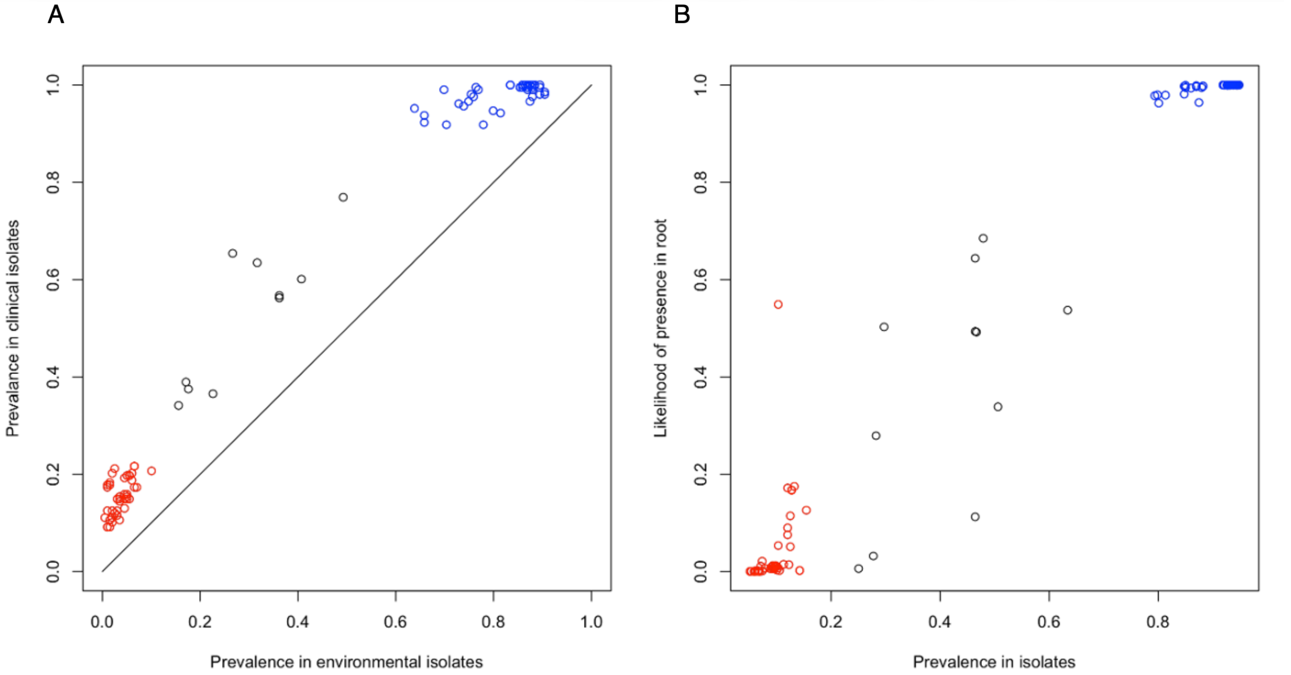

Supplementary Figure S11. Prevalence and ancestral state reconstruction of the 128 clinically enriched orthologs in *V. vulnificus.* A) Prevalence (presence in fraction of genomes) in environmental (x-axis) vs. clinical (y-axis) strains, where each data point is one ortholog. B) Prevalence among all 407 genomes (x-axis) vs. the likelihood that the ortholog was present at the root of the phylogenetic tree (in the last common ancestor of *V. vulnificus*) according to ancestral state reconstruction. The blue and red data points represent orthologs present in 91-100% and 9-22% of the clinical isolates, respectively.

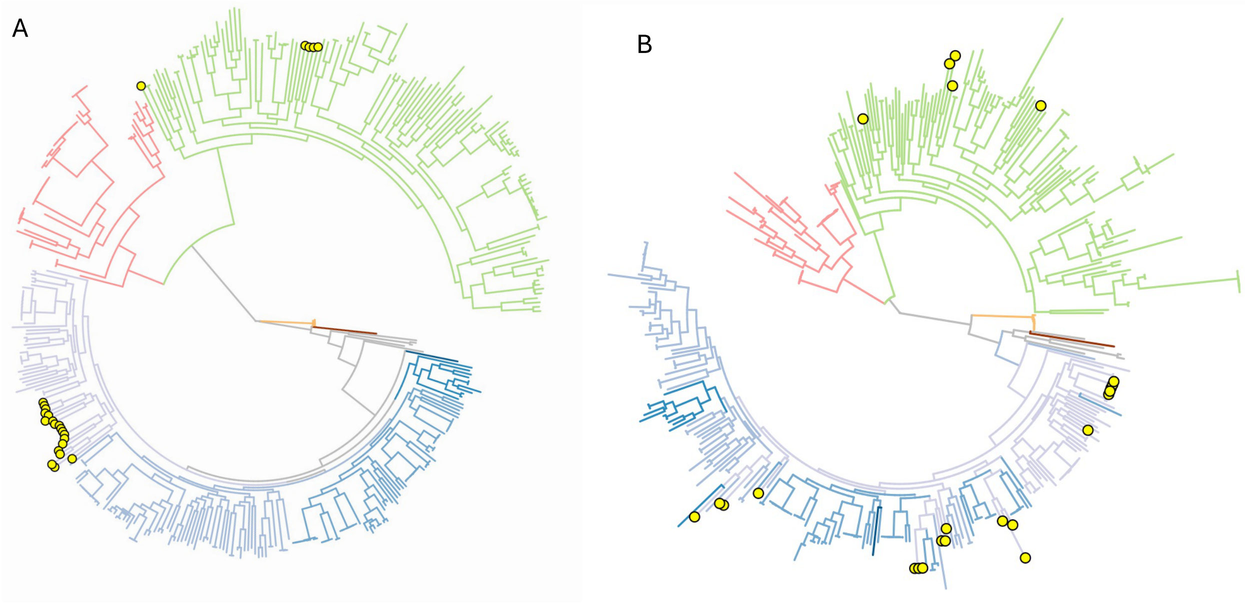

Supplementary Figure S12. Phylogenetic trees obtained with (**A**) the full core genome (as in Fig 2 and 4) and (**B**) with 86 orthologs located between the clinically enriched Clusters 5, 12, 11, 10 in a ~250 kb region on chromosome 2. The yellow points indicate the strains in which most of the genes of these clusters are missing. These strains are: S3_16, IRLE0056, CECT4608, 1676_80, Vv26, VA_WGS_18029, NV22, BaltVib12-S-24, BaltVib12-S-33, BaltVib12-S-41, VN_0206, BaltVib20-S-8, IRLA0043, VA_WGS_18037, VA_WGS_18038, BaltVib20-S-25, BaltVib20-S-49, BaltVib20-S-5, BaltVib20-W-65, SREL119, IRLE0062, SC9740, IRLE0057 and PV152.

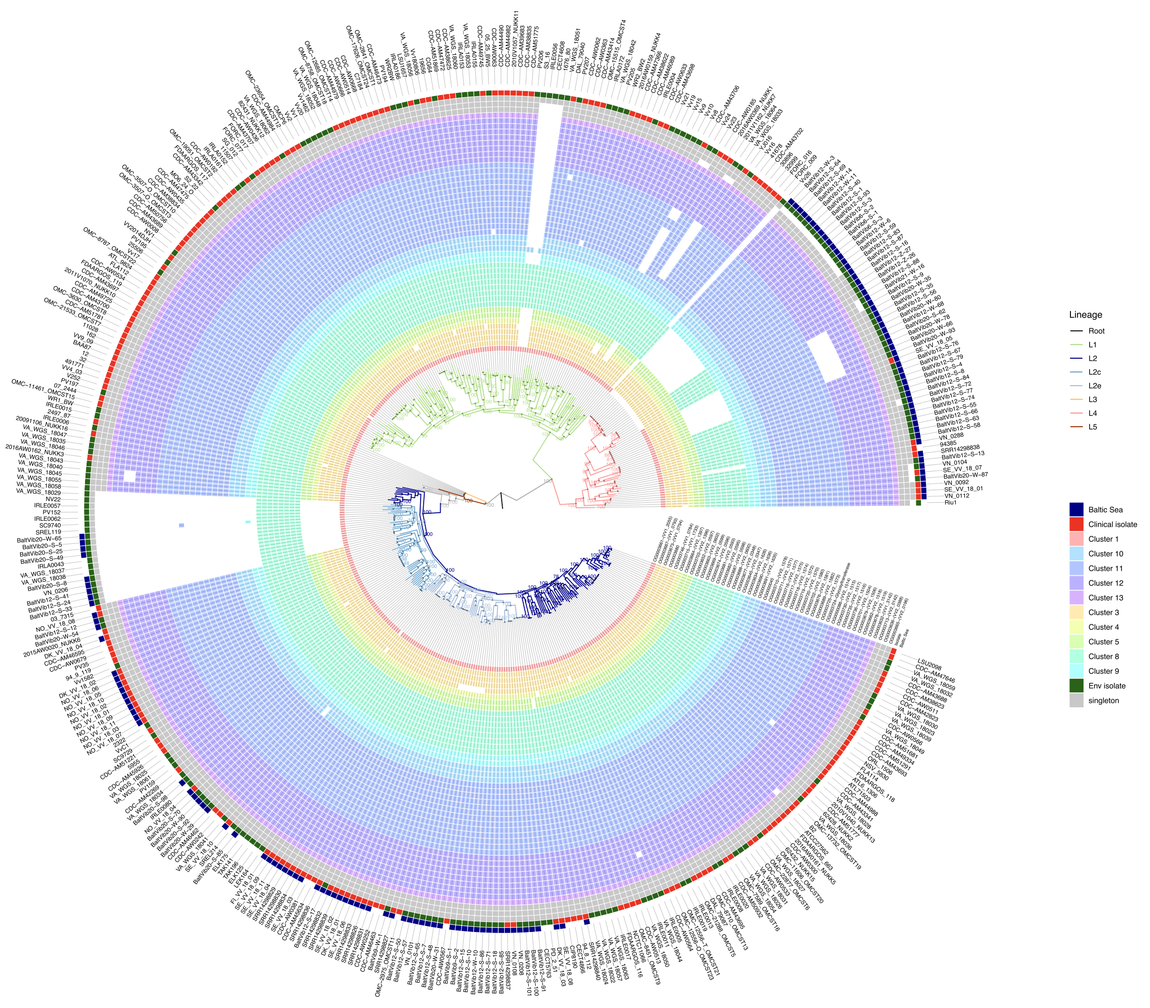

Supplementary Figure 13. Presence of the core clinically enriched orthologs in the 407 *V. vulnificus* genomes. The orthologs are ordered and colored according to co-localisation clusters.

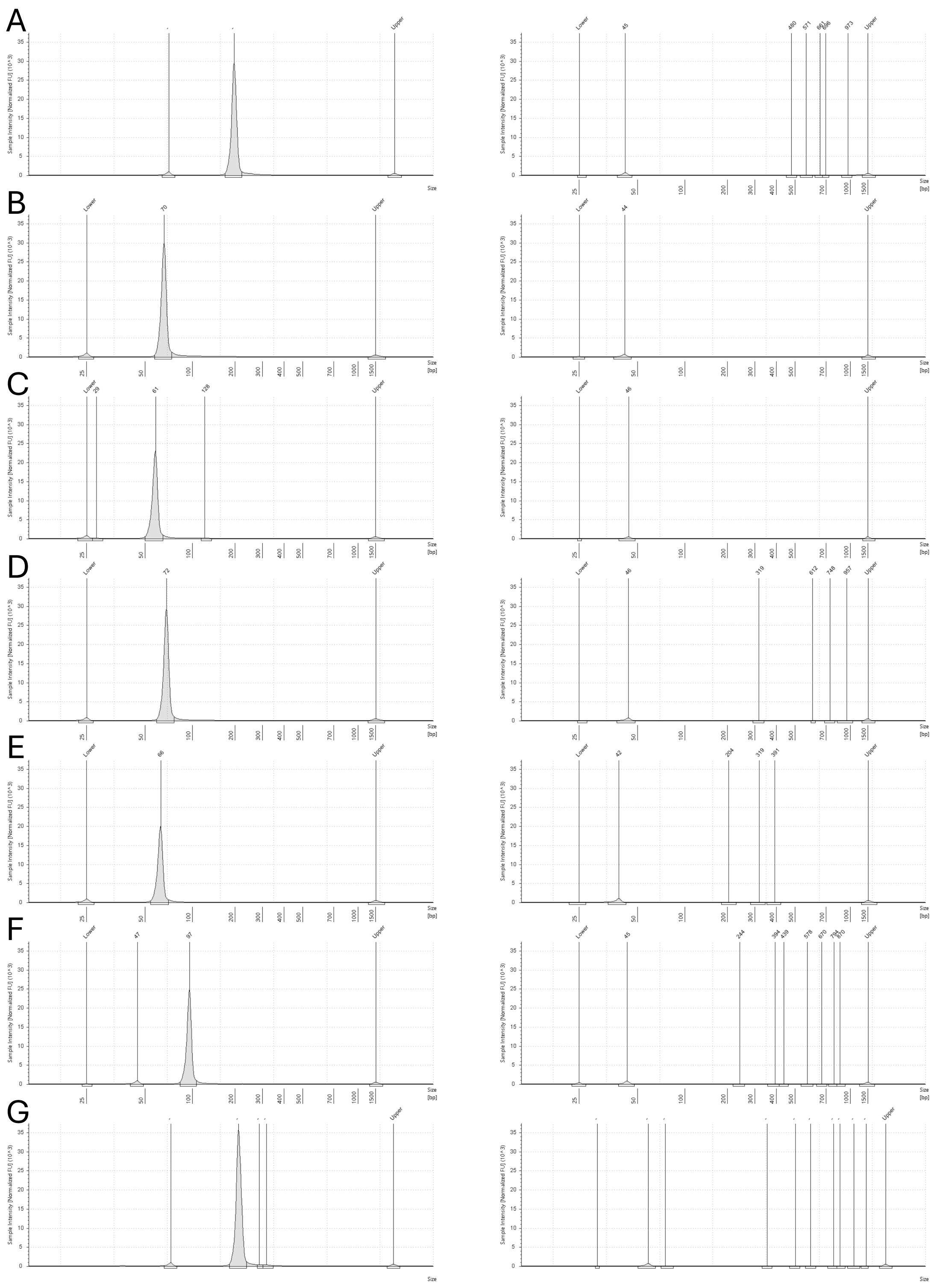

Supplementary Figure S14. Electropherograms of PCR products analysed on an Agilent TapeStation (High Sensitivity D1000 ScreenTape Assay). Panels to the left show results from PCRs run on DNA (0.2 ng per 20 µl reaction) from *V. vulnificus* ATCC 27562. Panels to the right show results from PCRs run on DNA (2.5 ng per 20 µl reaction) from Baltic Sea water sampled in spring (water temperature 3°). 30 PCR cycles were run. Primer pairs used: **A:** bap_F_1158 - bap_R_1233; **B:** cabA_F_189 - cabA_R_251; **C:** gly_F_824 - gly_R_875; **D:** dc_F_900 - dc_R_966; **E:** yjbf_F_229 - yjbf_R_287; **F:** ssd_F_39 - ssd_R_89; **G:** BaltVib_F_51 - BaltVib_R_56. For more information on the primers (including the applied annealing temperatures) see Supplementary Table 6. Faint signals are visible also in the water sample PCRs. Note however that these bands are clearly smaller (ca 45 bp) than those of the *V. vulnificus* PCR products. In **A** for *V. vulnificus* and **G** for both *V. vulnificus* and sea water, the lower size marker was not detected by the instrument and size could not be determined for the products. However, agarose gel analysis (data not shown) indicated that the sizes of the *V. vulnificus* PCR products were as expected also for these two primer pairs.
