# Supplementary material for: Phylogeny-aware comparative genomics of Vibrio vulnificus links genetic traits to pathogenicity
Source: mBio. 2026 Jun 17;17(7):e00205-26. doi: 10.1128/mbio.00205-26 (PMC13348674; doi:10.1128/mbio.00205-26)
Supplement: Legend — File S1 legend. [file mbio.00205-26-s0005.docx]

**Supplementary File 1:** Interactive map of the sampling locations, including the substations, from where the *Vibrio vulnificus* strains were isolates.
