# Supplementary material for: Phylogeny-aware comparative genomics of Vibrio vulnificus links genetic traits to pathogenicity
Source: mBio. 2026 Jun 17;17(7):e00205-26. doi: 10.1128/mbio.00205-26 (PMC13348674; doi:10.1128/mbio.00205-26)
Supplement: Fig. S4 to S6 — Presence and top 5 most common gene arrangements within co-localization Clusters 4, 12, and 5. [file mbio.00205-26-s0007.pdf]

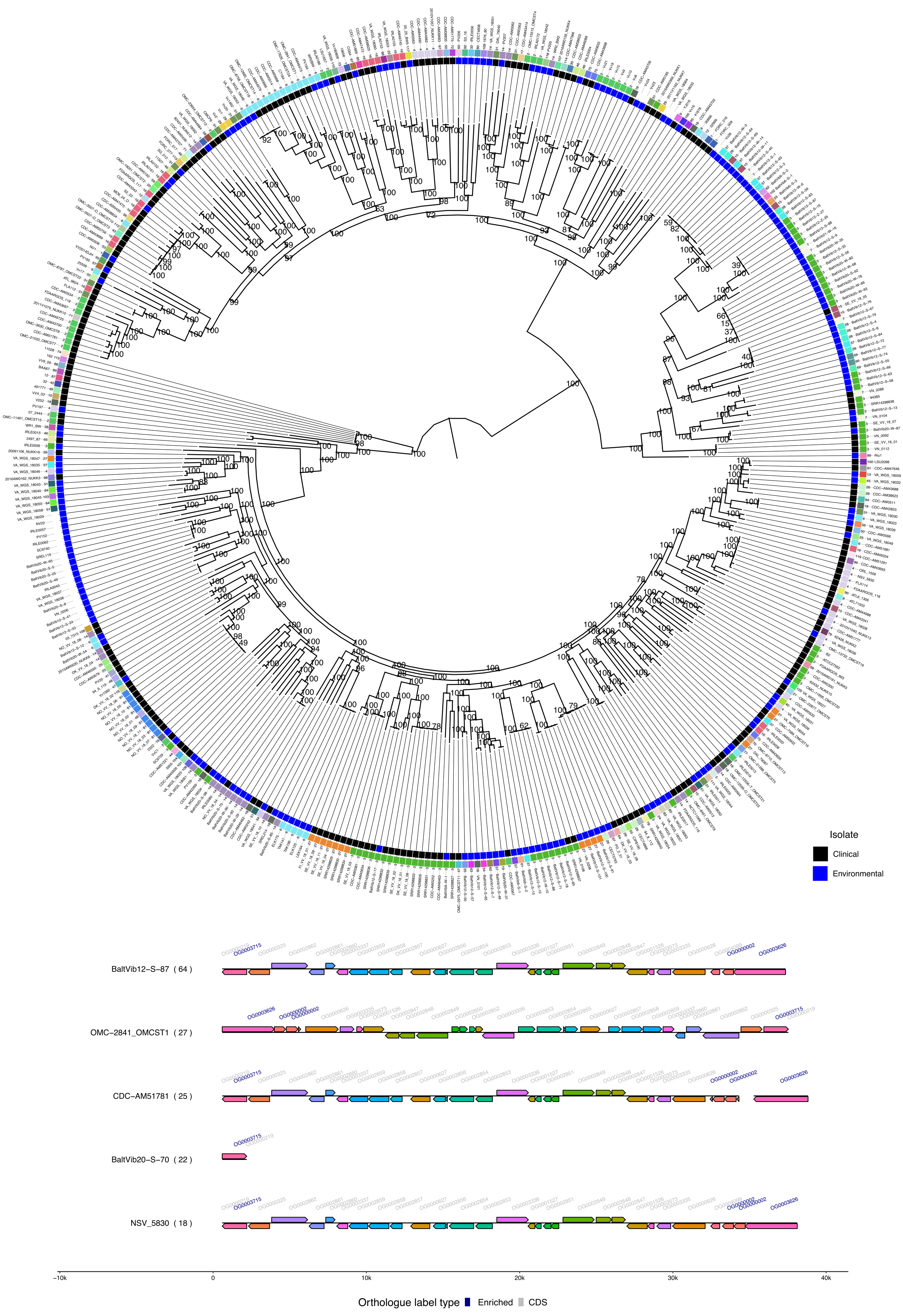

Supplementary Figure S4. Presence and top 5 most common gene arrangements within co-localization Cluster 4 in the 407 *V. vulnificus* genomes. Beside each gene arrangement, the ID of one genome and the number of genomes containing the arrangement are indicated. Orthologs with blue text are cluster members, grey text non-members. The leaf colors of the tree indicate the presence of different gene arrangements.

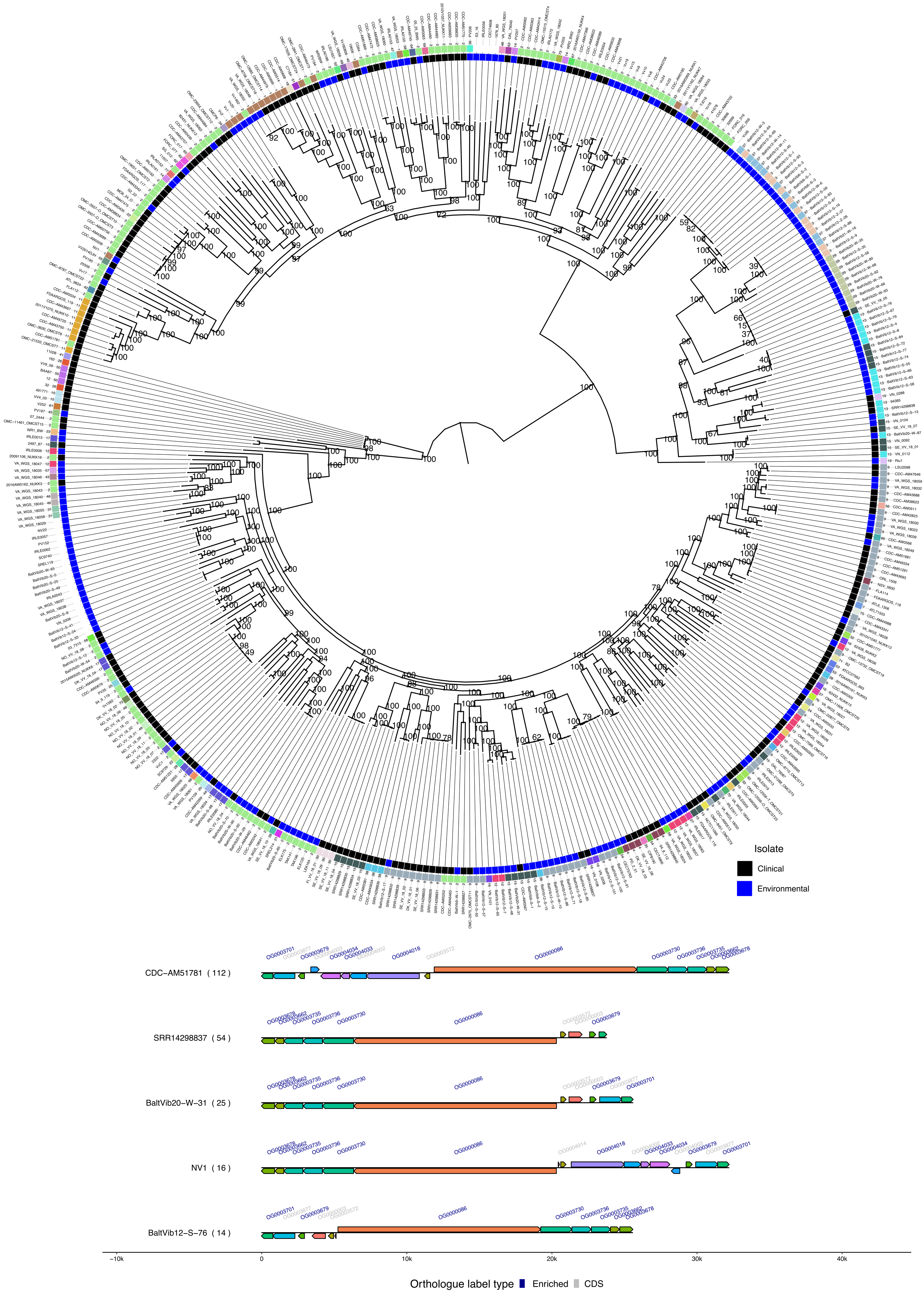

Supplementary Figure S5. Presence and top 5 most common gene arrangements within co-localization Cluster 12 in the 407 *V. vulnificus* genomes. Beside each gene arrangement, the ID of one genome and the number of genomes containing the arrangement are indicated. Orthologs with blue text are cluster members, grey text non-members. The leaf colors of the tree indicate the presence of different gene arrangements.

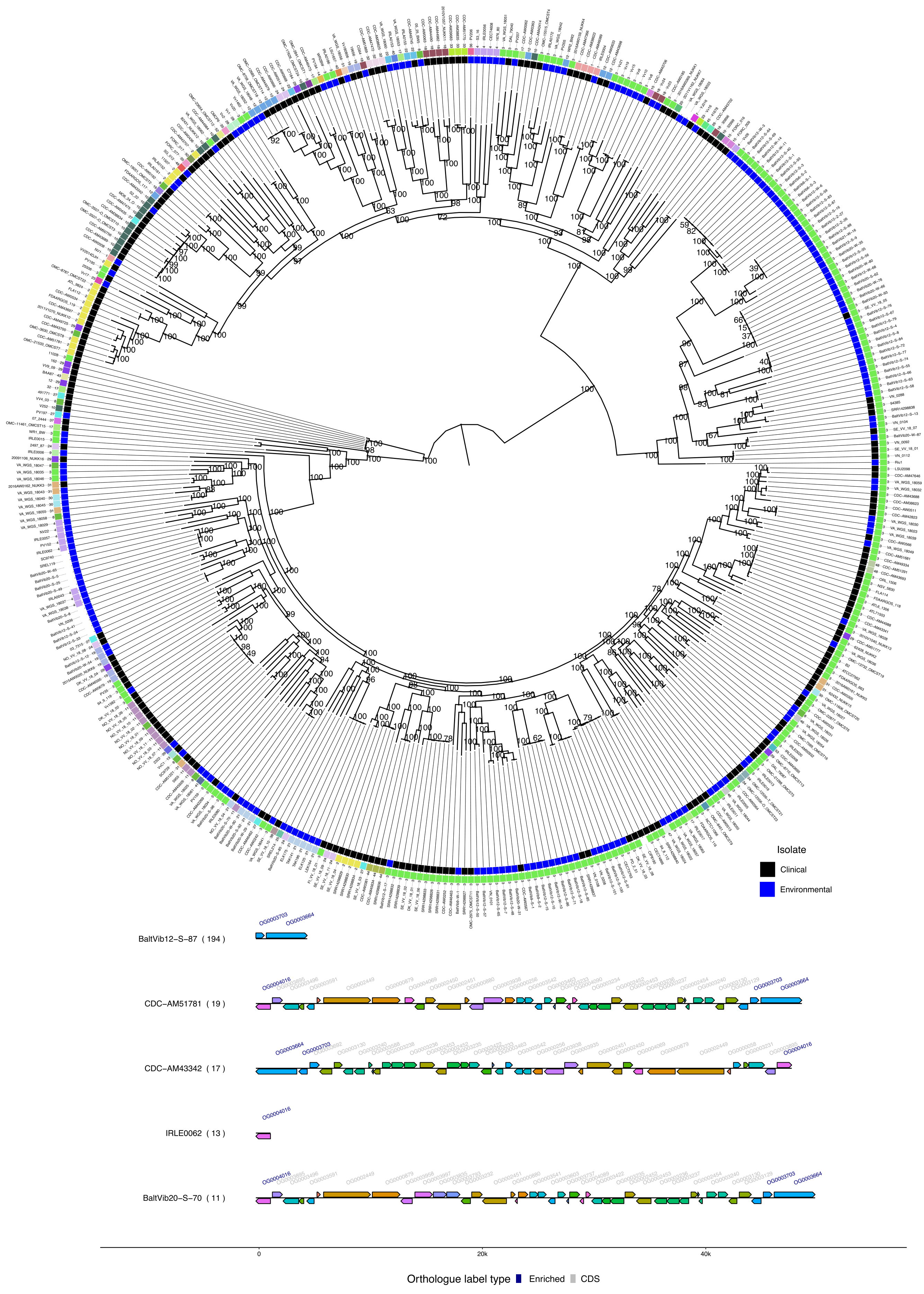

Supplementary Figure S6. Presence and top 5 most common gene arrangements within co-localization Cluster 5 in the 407 *V. vulnificus* genomes. Beside each gene arrangement, the ID of one genome and the number of genomes containing the arrangement are indicated. Orthologs with blue text are cluster members, grey text non-members. The leaf colors of the tree indicate the presence of different gene arrangements.
