# Supplementary material for: Phylogeny-aware comparative genomics of Vibrio vulnificus links genetic traits to pathogenicity
Source: mBio. 2026 Jun 17;17(7):e00205-26. doi: 10.1128/mbio.00205-26 (PMC13348674; doi:10.1128/mbio.00205-26)
Supplement: Fig. S7 to S9 — Presence and top 5 most common gene arrangements within co-localization Clusters 10, 15, and 13. [file mbio.00205-26-s0008.pdf]

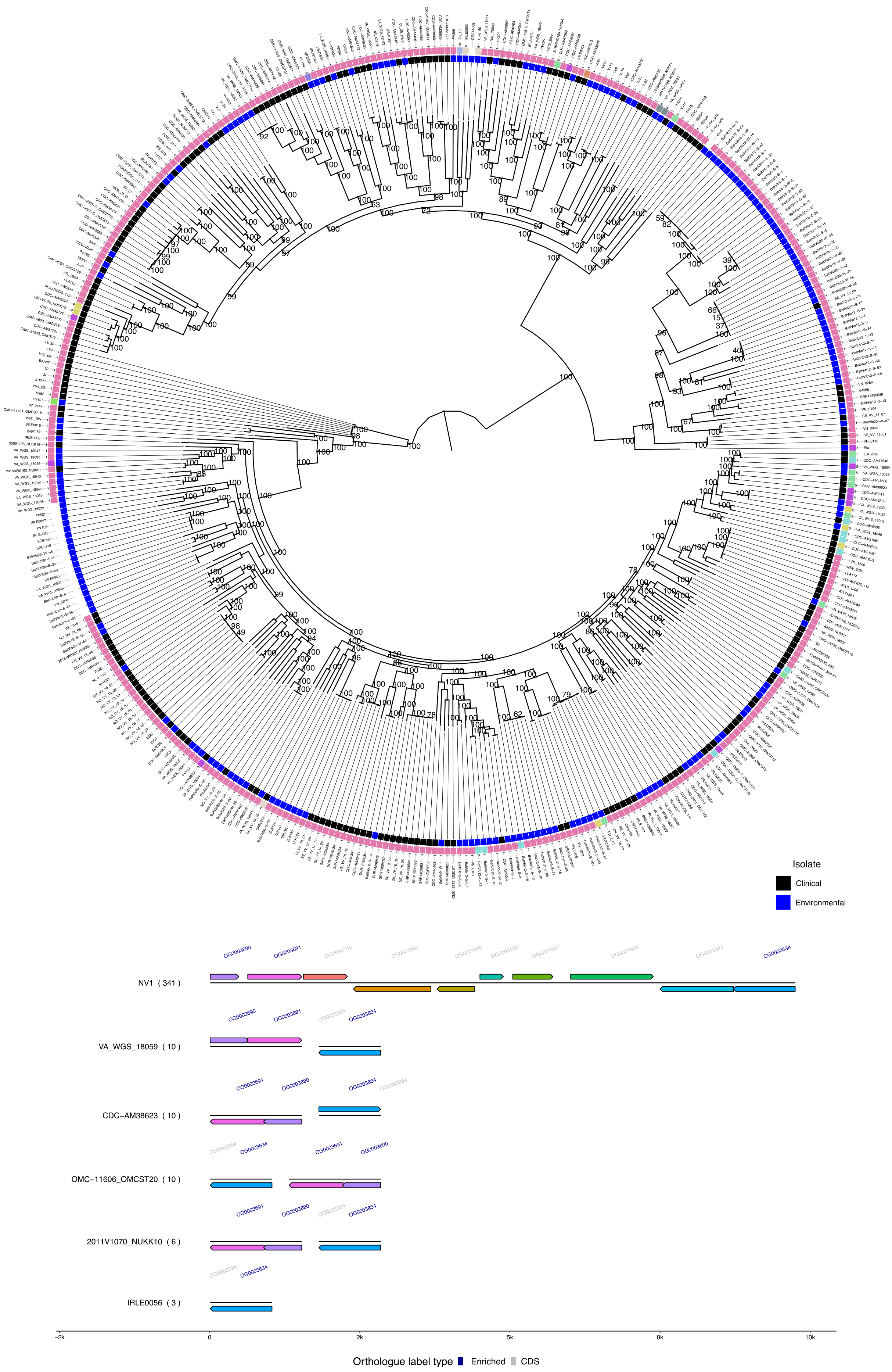

Supplementary Figure S7. Presence and top 5 most common gene arrangements within co-localization Cluster 10 in the 407 *V. vulnificus* genomes. Beside each gene arrangement, the ID of one genome and the number of genomes containing the arrangement are indicated. Orthologs with blue text are cluster members, grey text non-members. The leaf colors of the tree indicate the presence of different gene arrangements.

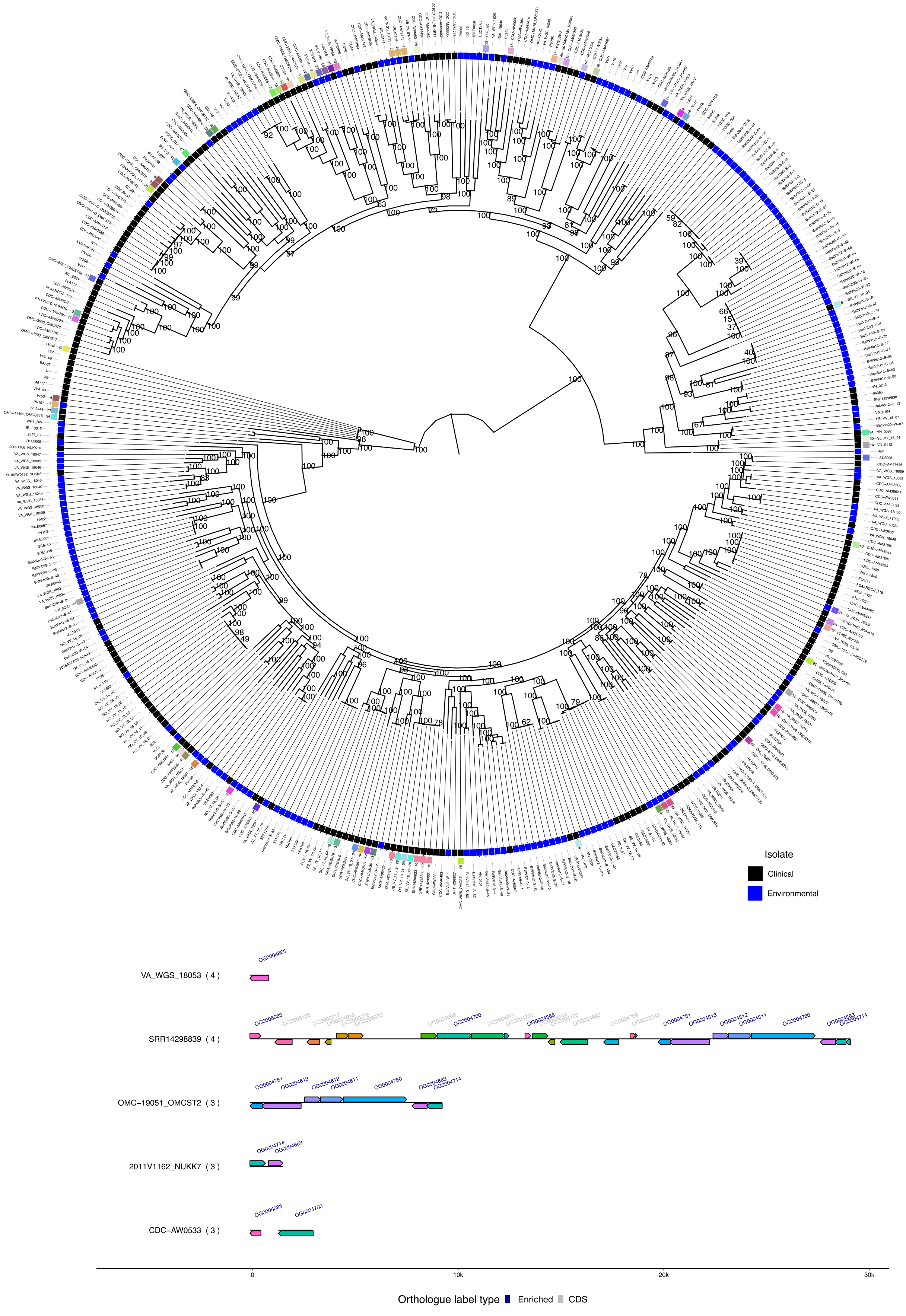

Supplementary Figure S8. Presence and top 5 most common gene arrangements within co-localization Cluster 15 in the 407 *V. vulnificus* genomes. Beside each gene arrangement, the ID of one genome and the number of genomes containing the arrangement are indicated. Orthologs with blue text are cluster members, grey text non-members. The leaf colors of the tree indicate the presence of different gene arrangements.

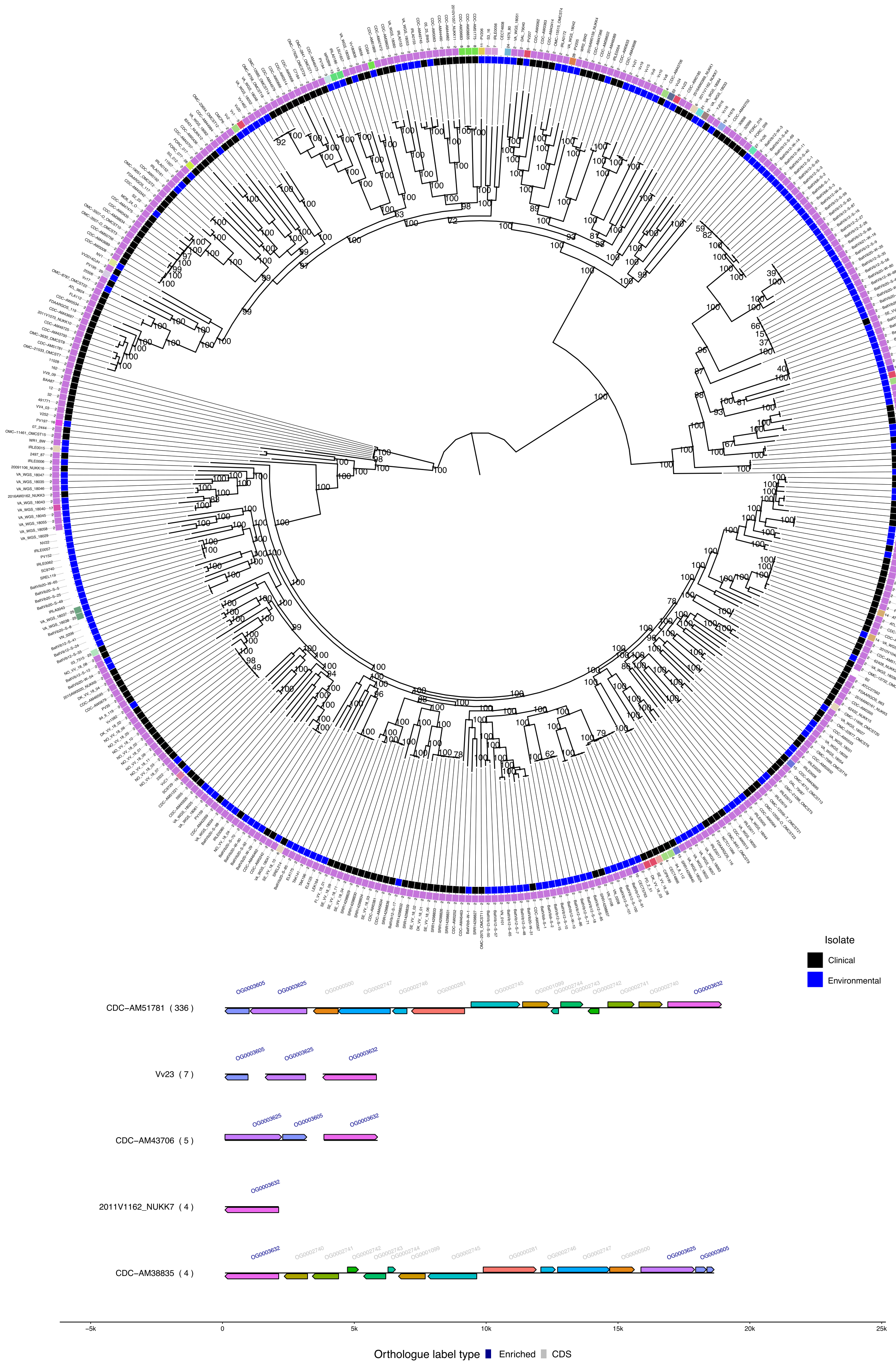

Supplementary Figure S9. Presence and top 5 most common gene arrangements within co-localization Cluster 13 in the 407 *V. vulnificus* genomes. Beside each gene arrangement, the ID of one genome and the number of genomes containing the arrangement are indicated. Orthologs with blue text are cluster members, grey text non-members. The leaf colors of the tree indicate the presence of different gene arrangements.
